# Effect of online intervention based on life skills for mental health, self-efficacy and coping skills among Arab adolescents in the Klang Valley, Malaysia: A cluster randomised controlled trial protocol

**DOI:** 10.1371/journal.pone.0298627

**Published:** 2024-02-23

**Authors:** Yosra Sherif, Ahmad Zaid Fattah Azman, Salmiah Md Said, Aishah Siddiqah Alimuddin, Hamidin Awang, Marjan Mohammadzadeh

**Affiliations:** 1 Department of Community Health, Faculty of Medicine and Health Sciences, Universiti Putra Malaysia, Serdang, Selangor, Malaysia; 2 Department of Psychiatry, Faculty of Medicine and Health Sciences, Universiti Putra Malaysia, Serdang, Selangor, Malaysia; 3 Psychiatry Unit, Faculty of Medicine and Health Sciences, Universiti Sains Islam Malaysia (USIM), Nilai, Negeri Sembilan, Malaysia; 4 Institute of Health and Nursing Science, Charité–Universitätsmedizin Berlin, Corporate Member of Freie Universität Berlin and Humboldt-Universität zu Berlin, Berlin, Germany; Institute of Biomedical and Health Research in Malaga (IBIMA), SPAIN

## Abstract

**Background:**

Migrant children and adolescents face a significantly increased risk of mental health issues. Focusing on this population’s mental health issues is fundamental and requires more attention to detect and reduce these burdens in adulthood. Nevertheless, life skills intervention can improve mental health. Its effects on Arab migrant adolescents have not been tested. Here, an evaluation protocol of the effect of an online life skills-based intervention for improving depression, anxiety, stress, self-efficacy, and coping skills among Arab adolescents in Malaysia will be examined.

**Material and methods:**

This cluster randomised controlled trial (RCT) will involve 207 Arab students (14–18 years old) from 12 Arabic schools in the Klang Valley. The schools will be assigned randomly to an intervention (online life skills programme) or control group at a 1:1 ratio. The researcher will deliver eight one-hour sessions to the intervention group weekly. The control group will receive the intervention at the evaluation end. Both groups will complete assessments at baseline, and immediately and three months after the intervention. The primary outcome is anxiety, depression, and stress [Depression Anxiety and Stress Scale-21 (DASS-21)]. The secondary outcomes are self-efficacy (General Self-Efficacy Scale) and coping skills (Brief COPE Inventory). Data analysis will involve the Generalised Estimation Equation with a 95% confidence interval. P < .05 will indicate significant inter- and intra-group differences.

**Discussion:**

This will be the first cluster RCT of an online life skills education programme involving Arab adolescent migrants in Malaysia. The results could support programme effectiveness for improving the participants’ mental health problems (depression, anxiety, stress), increasing their self-efficacy, and enhancing their coping skills. The evidence could transform approaches for ameliorating migrant children and adolescents’ mental well-being.

**Trial registration:**

The study is registered with the Clinical Trial Registry (Identifier: NCT05370443).

## Introduction

Mental disorders are one of the most frequent causes of disability and illness in children and adolescents [1, 2] and affect an estimated 10–20% of children and adolescents [3, 4]. More than 50% of all mental health problems begin before the patient is 14 years old and persist into adulthood [1, 5, 2]. Various factors can influence adolescents’ mental health, with early onset strongly linked to chronic issues, worse clinical outcomes, and lifetime repetition [6–8]. Among these factors contributing to poor mental health during adolescence are abuse and neglect, family conflict, violence, poor living conditions, chronic health problems and disabilities, stigmatisation and marginalisation due to gender identity and ethnic origin [6, 9–11].

There is an association between poor mental health and inferior physical well-being [6, 11]. Research shows mental health problems linked to adverse physical health conditions such as obesity, infection, and respiratory problems [12–15]. Children and adolescents with impaired mental health are also more likely to engage in unhealthy behaviours such as smoking, alcohol, and substance abuse, leading to poor physical health consequences [6, 11,]. Additionally, poor mental health can impact emotional and social development; for instance, poor academic performance and attendance at school, social isolation, and impeded cognitive development all have a long-lasting impact on physical health outcomes throughout a person’s lifetime [1, 11, 16]. Nevertheless, physical health problems can also influence mental health. Chronic pain, disability, and other issues related to various diseases can lead to anxiety and depression [3, 15, 17].

Impaired mental health in adolescents is also associated with various types of injuries, such as accidental injuries, including falls, burns, and drowning. This increased risk may be attributed to impaired judgment, impulsiveness, or poor decision-making skills. Their mental health issues can also lead to feelings of hopelessness, helplessness, and despair, which can elevate the risk of suicidal behaviour, suicidal ideation, and self-harm, resulting in injuries and a decline in the quality and length of life [18–21]. Moreover, adolescents with mental health issues face an increased risk of engaging in unsafe sexual behaviour, such as early sexual activity, unprotected sex, and having multiple partners, which can lead to adverse health outcomes. These experiences may also contribute to guilt, shame, and low self-esteem, exacerbating their mental health conditions [22, 23].

In the Eastern Mediterranean Region (EMR), which includes Arab children and adolescents, the estimated prevalence of mental disorders was between 10% and 36% [24], and most of the population in EMR countries is aged < 25 years [25]. Depressive symptoms were prevalent in 17.2% of adolescents of different nationalities living in the United Arab Emirates, with South Asian migrant adolescents recording the highest prevalence (33.3%), followed by Arab migrant adolescents (10.4%) [26]. In a 2009 study involving Saudi secondary school students, 13.9% and 14.3% had depression and anxiety, respectively [27], while increased depression and anxiety prevalence (56.3% and 56%, respectively) was recorded among secondary school students in Saudi Arabia in 2018 [28].

Over the past decades, many political, social, economic, and environmental stressors have affected Arab countries, and many Arabs travel and settle in other countries as either voluntary or forced immigrants [2, 29]. Arab adolescents encounter additional critical trials due to a range of social, cultural, and political factors that can negatively affect their mental health, for example, high rates of illiteracy, sharp educational disparities, with some countries struggling with inadequate resources, outdated curricula, declining education quality and gender inequality in education [9, 30–35]. Deficient health services and limited access to mental health facilities can make it difficult for Arab adolescents to access the care they need, leading to inadequate early intervention and treatment and deteriorating mental health status [9, 30].

The severe armed conflicts, violence, suppression, and economic inequalities in many Arab countries worsen these risk factors [30]. Furthermore, Middle Eastern Arab adolescents are generally afraid to seek help out of fear of appearing weak or embarrassing their parents and family [9]. Mental health issues are often stigmatised in Arab cultures, leading to delays in diagnosis and treatment and further exacerbating the condition. Cultural values and expectations among Arabs may strongly emphasise family honour, respect for authority, and conformity to traditional gender roles, which can create additional stress and pressure for adolescents and aggravate their mental health [9, 30, 31].

Migrant children and adolescents face a greater risk of anxiety and depression due to familial asymmetric acculturation, which occurs when individuals from different cultural backgrounds experience more pressure to acculturate, leading to an imbalance in the cultural adaptation and assimilation process. For example, migrants to a new country may experience pressure to learn the language and cultural norms of the host culture [36, 37]. In addition, lack of adequate parental support, obstructive practises due to discrimination, poor socioeconomic station, residence in crowded homes and settlements in city districts with inadequate organisation, and inadequate healthcare system access [38–41].

In the United States, 14% of Arab adolescents had moderate or moderately severe depression [42], and 80% had not told anyone or sought help as mental illness is stigmatised, which may exacerbate the condition [40, 42], where they faced an increased discrimination risk and acculturational stress. Therefore, early intervention is critical for reducing mental health issues among Arab migrants. It enables the timely identification and treatment of mental health problems, preventing them from becoming more severe and persistent [1, 6, 11]. Nonetheless, there is a much smaller evidence base for Arab migrants’ mental health due to the scarcity of research. Accordingly, it is essential to focus on this area and highlight it.

Systematic reviews have demonstrated the importance of school-based interventions for reducing depression, anxiety, and other mental disorders [6]. Using the school system as the background for implementing prevention programmes could present a significant opportunity for students because the schools provide a unique and vital setting for identifying and addressing mental health concerns by early discovering mental disorders or delaying their onset. Schools offer regular contact with students, which allows for ongoing observation and monitoring of their behaviour and emotional well-being. Schools can also play a role in reducing the stigma associated with mental health disorders. By providing education and support around mental health, schools can help create a more accepting and supportive environment for students struggling with mental health concerns. Therefore, school curriculum-integrated programmes could also assuage many representative barriers to accessing treatment, such as location, time, and cost [43, 44].

Life skills (interrelated components that act in combination and reinforce one another) are described by the WHO as "abilities for adaptive and positive behaviour that enables individuals to deal effectively with the demands and challenges of everyday life" [45]. Life skills are necessary for overcoming the many obstacles adolescents face and help develop self-efficacy, self-esteem, psychosocial competence, and comprehensive self-development. Life skills enable individuals to avoid potentially harmful actions while maximising the effectiveness of other crucial protective variables [46] and can be developed through well-designed interventions.

A life skills programme can enhance mental health and psychological well-being [47–50]. Therefore, it is essential to develop and design programmes that educate and support a diverse range of teenagers who may be at risk for developing mental health difficulties, especially those with migrant backgrounds and who are susceptible to developing mental health issues [4]. Life Skills Programme is an educational intervention aimed at improving mental health and boosting adaptive and positive behaviour in the target individuals [51, 52]. Generally, the life skills curriculum involves skills strongly connected to the essential elements that promote youth resilience and healthy emotional outcomes [52, 53]. The life skills programme significantly reduced youth behavioural problems and increased self-efficacy [54]. Individuals with high self-efficacy (belief in their abilities) are more likely to cope effectively with stressful situations to reach their goals and have a higher subjective well-being level and experience positive impact and satisfaction with life [55].

### Theoretical and conceptual frameworks

The proposed life skills programme in this study will be based on the social cognitive theory developed by Albert Bandura, who focused on the cognitive aspects of observational learning and how behaviour, cognition, and the environment interact to shape individuals. Bandura introduced the principle of the dynamic and shared relationship between a person, their environment, and their behaviour [56]. Acquiring life skills relies on active engagement, where the training is organised to provide practise opportunities in an encouraging learning environment [45].

The social cognitive theory emphasises learning by observing others. Numerous fundamental assumptions influence the social cognitive view. First, students can learn by watching others who serve as role models and develop new knowledge and behaviours by following these role models, who can be real people or characters in media. By observing and imitating the actions of these models, individuals can acquire new skills, knowledge, and behaviours that they may not have learned through direct experience or instruction. There is also an assumption of goal-directed behaviour, where it is hypothesised that students create goals, direct their behaviour appropriately, and are inspired to achieve those goals. Moreover, their behaviour ultimately becomes self-regulated via continual corroboration from exterior encouragement to improve, which ultimately improves the students’ mental health [45, 56].

Based on the theory, life skills education would enhance the student’s belief in their ability and capabilities for creative thinking, problem-solving, effectual communication, managing stress; self-control via goal-setting, self-monitoring, self-reward, feedback; empathy, positive social skills, and knowledge to implement new behaviours by exposure to interpersonal demonstrations mainly via peer modelling and develop their mental health status through all of this [45, 46].

In this study, the critical concept of a life skills programme is suggested and designed to recover mental health status with the ultimate goal of changing the anxiety, depression, and stress scores in Arab students in Malaysia and increasing their self-efficacy and coping skills. The students will observe the behaviour of others in their social circles and develop an idea of how new actions are performed. This recorded information will guide action on subsequent occasions.

## Materials and methods

### Study aims and objectives

- To determine the effects of online life skills-based intervention on reducing depression, anxiety, and stress between the intervention and control groups and within the intervention groups immediately and three months after the intervention, and after adjusting for covariate variables.
- To determine the effects of the intervention on improving self-efficacy and coping skills between the intervention and control groups and within the intervention groups immediately and three months after the intervention and after adjusting for covariate variables.

### Study hypothesis

- The mean scores for depression, anxiety, and stress will show significant reductions in the intervention group compared to the control group (between groups). These reductions will also be observed within the intervention group immediately, three months after the intervention compared to the baseline scores (within groups), and after adjusting for the covariates.
- The mean scores for self-efficacy and coping skills will show significant differences in the intervention group compared to the control group (between groups). These differences will also be observed within the intervention group immediately, three months after the intervention, compared to the scores before the intervention (within groups), and after adjusting for the covariates.

### Study design

**Fig 1:**
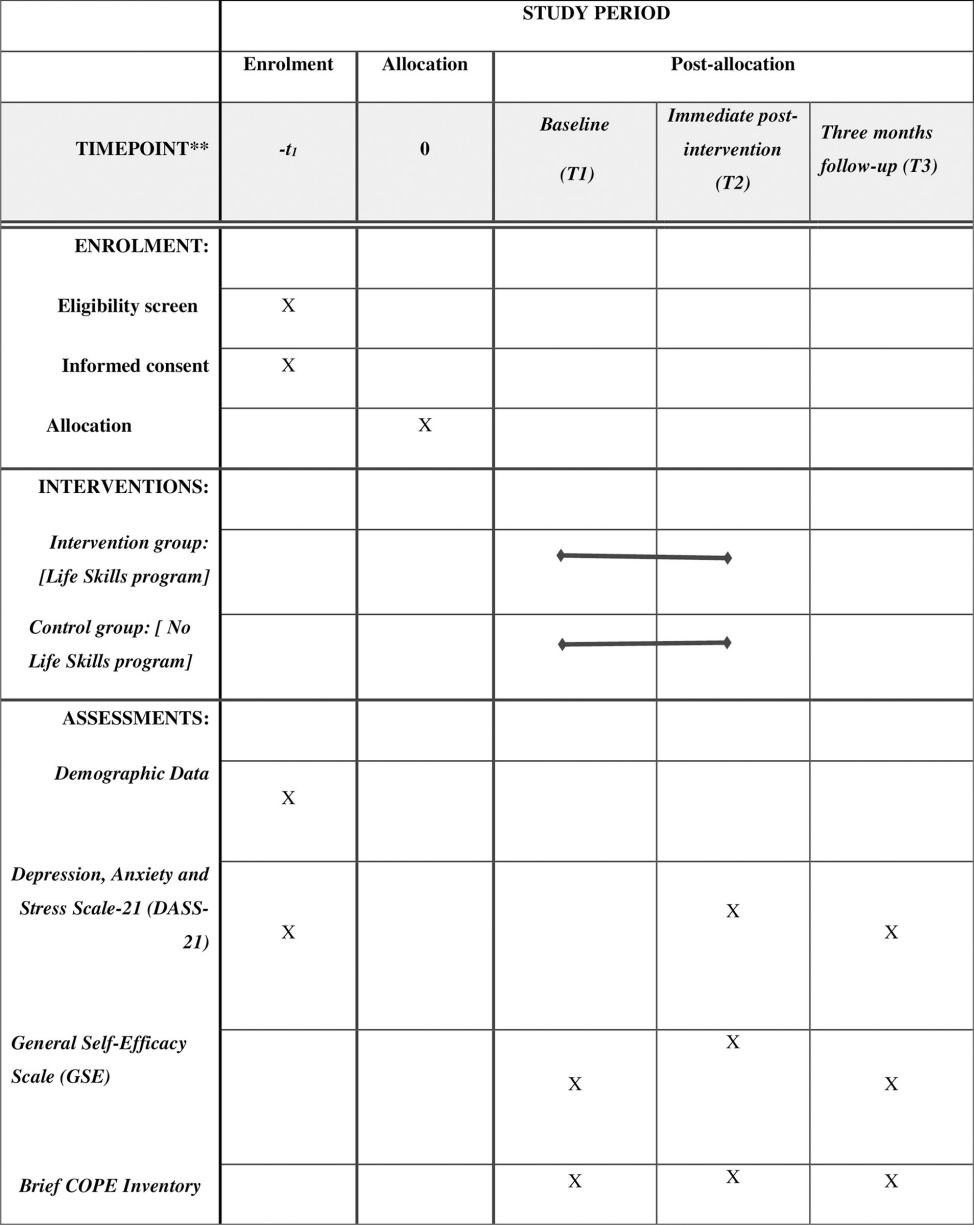
SPIRIT schedule of enrolment, interventions, and assessments [57].

This will be a parallel cluster randomised controlled trial (RCT) that follows the Recommendations for Interventional Trials (SPIRIT) (**Fig 1**) [57] and Consolidated Standards of Reporting Trials (CONSORT) guidelines and CONSORT statement [58]. The Cluster Randomized Controlled Trial (RCT) design was chosen to minimise the possibility of intervention contamination. Additionally, this design is more feasible and efficient regarding resources, as online interventions can be simultaneously delivered to multiple individuals [59]. The SPIRT checklist and study protocol are provided in Supporting Information as S1 Checklist and S1 File.

**Fig 2:**
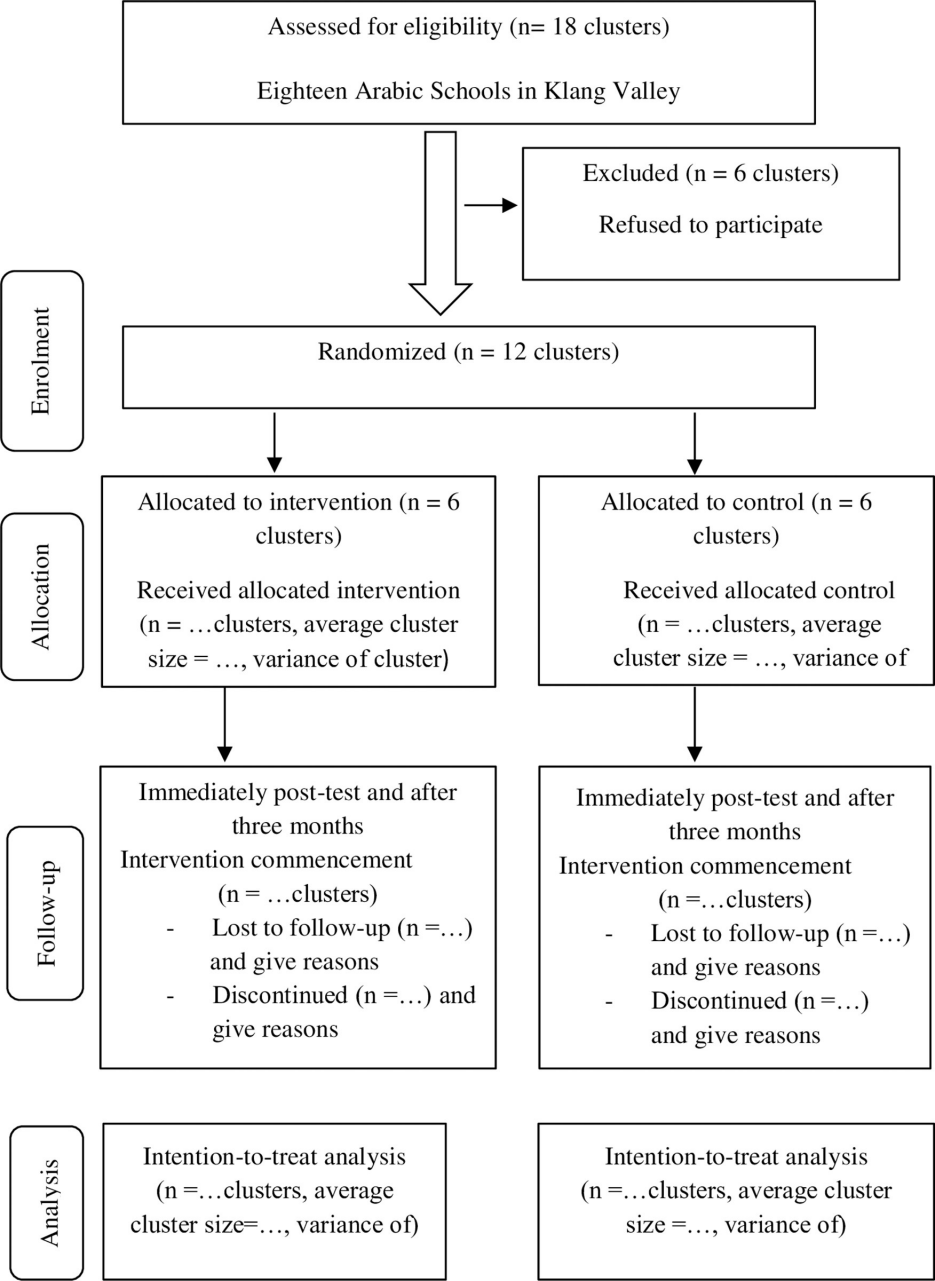
CONSORT flow diagram of the study adapted from [58].

This study consists of a screening phase and a trial phase. In the screening phase, eligible students with mild to extremely severe depression, anxiety, and stress levels will be identified using the Depression, Anxiety and Stress Scale-21 (DASS-21) questionnaire. In the trial phase, the intervention effectiveness in reducing the participants’ depression, anxiety, and stress levels will be assessed (**Fig 2**).

### Study location

The study will be implemented in Arabic schools in the Klang Valley, Selangor, where the Arab population in Malaysia is highest. There are approximately 18 Klang Valley Arabic schools, most of which teach Arab students at primary, and lower and upper secondary education levels. The schools feature a similar education system structure to that in Malaysia and consist of six years of compulsory primary education from ages 6–11 years and three years each of lower and upper secondary education. Subsequently, the students are required to pass a General Certificate of Secondary Education examination to enter university.

### Recruitment of Arabic schools and participants

**Fig 3:**
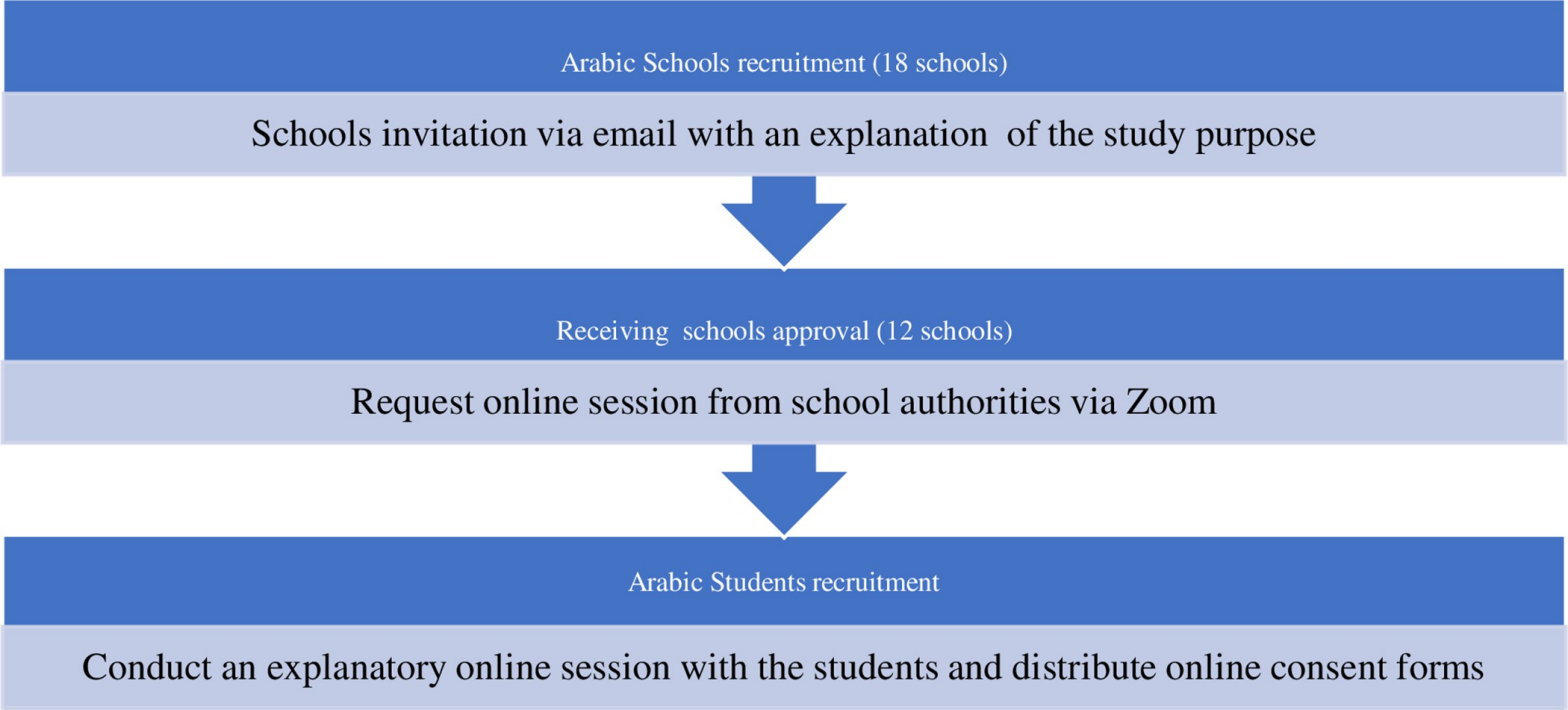
Recruitment of Arabic schools and participants.

The recruitment consists of two stages: firstly, the researchers have prepared a list of all Arabic schools in Klang Valley and sent invitation emails for them to participate in the study. Six of the 18 Arabic schools invited to the study were excluded as they declined to participate. The remaining 12 schools, with 648 students aged between 14 and 18 years, were recruited to participate in the research and will be assigned to the control or intervention arms. Secondly, a small online meeting group will be arranged with each school via the Zoom platform in coordination with the school principals. During the meeting, the researcher will describe the purposes and benefits of the study and the criteria for the inclusion and exclusion of the participants **(Fig 3)**. Accordingly, written informed consent will be obtained from the students and parents who agree to participate. The participants will be required to complete baseline data questionnaires for screening. Following the control or intervention treatment of eligible students selected from each school, the participants will be required to complete the same questionnaire set (except the personal information form) after the intervention and in the follow‐up evaluation.

### Inclusion and exclusion criteria

Arabic schools will be eligible for participation if: (1) they agree to participate and 2) are in the Klang Valley. The exclusion criteria are Arabic schools that decline to participate in the study and non-Arabic schools with Arab students. The inclusion criteria for students are as follows: (1) Arab students aged 14–18 years; (2) who assent and have their parents’ consent; (3) mild to extremely severe depression, anxiety, and stress scores based on the baseline assessment (screening phase). The exclusion criteria for students are as follows: (1) refusal to participate; (2) hearing limitations as they would be unable to interact during the online session; (3) previous diagnosis or treatment for mental health problems based on their school file may act as a confounding factor and interfere with the effect of the programme.

### Ethical considerations

Ethical clearance to conduct the study was obtained from the Universiti Putra Malaysia ethical committee [JKEUPM-2021-912] (S1 Appendix). The directors of the Klang Valley Arabic schools have granted their permission for participation. All participants and their parents will be informed of the study purposes, data collection time, and possible risks and benefits prior to their involvement in the study. The researcher will obtain written assent from all participants and consent from their parents (S2 and S3 Appendices). Data will be stored confidentially and anonymously, and no third party will have access or visibility to the data. It will be stored in a password-protected file on a password-protected encrypted computer. In case of an emergency or if any participant feels stressed or anxious by the programme or their existing condition(s) worsens, they will be released from the study and referred to healthcare professionals for further management and evaluation after discussion with their parents. This study is registered at ClinicalTrials.gov (Identifier: NCT05370443).

### Sample size calculation

The sample size was calculated based on the equation of two independent population means [60, 61]. The sample size necessary for a parallel cluster RCT was determined with G*Power 3.1 software, which yielded N = 118 based on the following: M1 (S1) = mean (M) and standard deviation (S) of depression score of intervention group participants who completed the programme (post-intervention) = 7.06 (3.20), M2 (S2) = mean and standard deviation of depression score of control group participants who completed the programme (post-intervention) = 8.79 (3.43) [62], moderate effect size of 0.50, alpha of 0.05, power of 0.8, and a 1:1 ratio for the two groups. By considering the intra-class correlation coefficient (ICC, a statistical measure of the degree of outcome correlation within clusters), this estimate was increased using the design effect [1 + (n– 1) ρ, where n = average cluster size and ρ = ICC].

The assumed average cluster size was 20 participants per school, and the ICCs were 0.02 [63]. This yielded a design effect of 1.4, which was multiplied by the original estimated sample size of 118. After adjusting for the attrition rate and multiplying by the design effect, the adequate sample size was 207, which was the total sample size required. The sample size calculation determined that 10 schools will be required. Nevertheless, to obtain the required sample size, the 12 schools that met the inclusion criteria will be included [64].

### Sampling method

The 12 aforementioned Klang Valley Arabic schools will be included in the study. Following cluster sampling, the intervention and control groups will comprise six schools each. To obtain the required sample size, all students in the included schools who grant permission and provide their parents’ consent to participate will be included in the first study stage. Subsequently, they will be required to complete the questionnaires (screening). Students with a minimum mild score for at least one aspect of the DASS-21 (depression, stress, or anxiety) will be eligible for inclusion in the intervention study.

### Randomisation and allocation concealment mechanism

Arabic schools will be considered the unit of randomisation and randomised into two groups to avoid contamination. The schools will be allocated randomly based on a 1:1 ratio to the intervention and control groups to maintain an approximately equivalent number of schools among the two groups. An independent statistician will conceal the allocation by generating the allocation sequence list with block randomisation software and randomly block sizes. Each cluster will be assigned a distinctive software-generated code in a closed opaque envelope. Subsequently, the envelopes will be opened by a team assistant who will assign the clusters to the intervention or control group based on the codes. Lastly, the students will be assigned to the intervention and control groups. No control group participant will be aware of what the intervention group will be offered. Furthermore, the group allocation will not be known to the statistician who will perform the primary analyses.

### Intervention

#### Intervention development

The educational intervention will be guided by the social cognitive theory. In consultation with experts in mental health, life skills, and education programme development, the intervention will be adapted from the WHO Life Skills Education Programme and UNICEF (United Nations Children’s Emergency Fund) guidelines for implementing life skills [45, 51, 52]. Overseen by the supervisory committee, the modules will be developed based on the research objectives, Arab culture, the target population, study duration and limitations, and the pilot study results. The intervention will enhance the participant’s mental health and assist them in decreasing anxiety, depression, and stress and increasing self-efficacy and coping skills. This programme will also be designed to improve the participant’s ability to cope with life challenges during migration and after settlement in a new country. Important considerations, such as those related to Arabic culture during programme module development and the problems most commonly face by Arab migrants in Malaysia, will be taken during the preliminary activity development and design, as they will be designed to directly emphasise managing external stressors and emotions.

#### Intervention content

The sessions will target self-awareness, empathy, interpersonal relationship skills, communication skills, creative and critical thinking, decision-making, problem-solving, and managing stress and emotion. Concept videos, brainstorming, storytelling, case study, small-group discussions, real-world skills practice, and real-life situation simulation will be used (see **Table 1**).

**Table 1:** Overview of the intervention on improving self-efficacy, mental health, and coping skills.

Topics	Life skill	Target	Type of materials
1. Introduction of life skills, myself as a person, rights and responsibilities	Self-awareness	Mental health and self-efficacy.	Education videos, Discussion sessions, Real-life situation simulations. case story, real-world skills practice
2. Understanding interpersonal similarities and differences and how to appreciate such differences	Empathy	Mental health and self-efficacy.	Education videos, Discussion sessions, Real-life situation simulations, case story, and real-world skills practice
3. Learning the importance of relationships with family and friends	Interpersonal relationship skills	Mental health, and self-efficacy.	Education videos, Discussion sessions, Real-life situation simulations, case story, and real-world skills practice
4. Basic verbal and non-verbal messaging skills + confident messaging to face peer pressure	Communication skills	Mental health, and self-efficacy.	Education videos, Discussion sessions, Real-life situation simulations, case story, and real-world skills practice
5. Learning basic critical thinking processes	Critical thinking	Mental health, self-efficacy, and coping skills.	Education videos, Discussion sessions, Real-life situation simulations, case story, and real-world skills practice
6. Developing capacities for thinking creatively and generating novel concepts	Creative thinking	Mental health, self-efficacy, and coping skills.	Education videos, Discussion sessions, Real-life situation simulations, case story, and real-world skills practice
7. Learning basic and advanced steps for problem-solving and decision-making	Problem-solving and Decision-making	Mental health, self-efficacy, and coping skills.	Education videos, Discussion sessions, Real-life situation simulations, case story, and real-world skills practice
8A. Identifying causes of stress and methods for managing stressful circumstances 8B. Recognition of the expression of different emotions and coping with emotional distress	Coping with stress and emotions	Mental health, self-efficacy, and coping skills.	Education videos, Discussion sessions, Real-life situation simulations, case story, and real-world skills practice

### Intervention implementation

Forty-eight hours after the baseline data are collected, the researcher will conduct at least one-hour programme per session via Zoom. However, the duration of the sessions dedicated to each topic may vary depending on the needs of the students. In some cases, each topic may be covered in one hour, while in other cases, several hours may be devoted to each topic to allow for more in-depth exploration and skill-building. The sessions will be carried out weekly with a small group of students for eight consecutive weeks. The intervention will be implemented every Saturday with the help of school coordinators so that it will not interfere with the student’s academic schedules. The intervention will be implemented at different times for each school in the intervention group with the same quality. Before the intervention commences, all participants will receive a short reminder text message to enrol in the online session and subsequently watch the video.

Online platforms offer a more cost-effective and convenient way to provide training and counselling that aligns with the lifestyle of Arab adolescents in Malaysia. This is particularly relevant after the COVID-19 pandemic, during which all Arabic schools in Malaysia conducted virtual classes, and most Arab adolescents are familiar with online platforms. Nonetheless, implementing traditional face-to-face interventions in schools can pose challenges due to their high resource requirements in time and money. Alternatively, online interventions have greater accessibility, convenience, and flexibility.

### Evaluation of the intervention

The follow-up evaluation will be performed immediately and three months after the intervention for all students in both groups. The students will be assessed by an online survey via Google Forms. The contents of the immediate and three-month post-test assessments will be equivalent to that of the baseline assessment, except that the sociodemographic questions will be excluded from the post-test assessments. The differences between and within the two groups for the depression, anxiety, stress, self-efficacy, and coping skills mean scores will be assessed with the appropriate statistical tests.

### Intervention module validity

#### Content validity

The content validity of the modules will be checked and assessed by a panel of professional experts from the Community Health Department and Psychiatric Department of the Universiti Putra Malaysia Faculty of Medicine and Health Science, and experts in life skills education with research experience involving life skills. The experts will check the educational material and assess whether it is accurate, efficient, and appropriate considering the objectives. Their comments and suggestions will be incorporated into the final life skills modules.

#### Face validity

Some activities from the modules will be chosen randomly to examine the face validity via a sample of 10 Arab students aged 14–18 years from Klang Valley Arabic schools that are not participating in the main study. The comprehensibility, clearness, and quality of the activities and modules will be examined. Subsequently, unfitting or challenging-to-comprehend phrases will be identified and modified based on the students’ comments.

### Control

The control group participants will not receive life skills education during the study period. Nonetheless, they will be assigned the same educational activities after completing the evaluation at the study conclusion. They will be monitored as part of the study and required to answer the same questionnaires at baseline, immediately after, and three months after the intervention for comparison with the intervention groups.

### Outcome measures

A validated Arabic version of sociodemographic questionnaires, the Depression Anxiety and Stress Scale (DASS-21), the General Self-Efficacy Scale (GSE), and the Brief COPE (Coping Orientation to Problems Experienced) Inventory (study outcomes) will be used in this study. The developers of the aforementioned questionnaires waived the requirement for permission if the questionnaires are used for research purposes.

#### Sociodemographic variables

Gender, age, nationality, student’s and parents’ education level, duration of residence in Malaysia.

#### The DASS-21

The DASS-21 is the short version of the DASS-24 developed by Lovibond and Lovibond [65]. The DASS-21 comprises three self-report scales designed to measure the negative emotional states (depression, anxiety, and stress). Each scale contains seven items with a four-point Likert scale (0 = does not apply and 3 = very applicable or applies most of the time). The Arabic-version DASS-21 has been validated in adolescents (Cronbach’s alpha coefficients for anxiety, depression, and stress = 0.73, 0.75, and 0.73, respectively [66–68].

#### The GSE

The GSE is a 10-item psychometric scale rated on a four-point scale that has scores ranging from 10 to 40. The GSE is prepared for respondents aged ≥ 12 years and is designed to assess the strength of a person’s belief in their ability for responding to new circumstances, difficulties, or life stresses. The internal consistency of the GSE is high, with Cronbach’s alpha between 0.75 and 0.91, and all questionnaire items were loaded on a single component [69]. The total Cronbach alpha coefficient of the Arabic-version GSE is 0.75 [70].

#### Brief COPE inventory

The Brief COPE Inventory that will be used in this study consists of 28 items classified into four categories scored from one ("I haven’t been doing this at all") to four ("I’ve been doing this a lot") that explore 14 strategies: self-distraction, active coping, denial, substance use, use of emotional support, use of instrumental support, behavioural disengagement, venting, positive reframing, planning, humor, acceptance, religion and self-blame. It includes two items related to each strategy. The items will be totalled on a four-point Likert scale, and the total score will be calculated for all four categories. Higher scores indicate a higher propensity to apply the resultant coping strategies [71, 72].

#### Data collection

The questionnaires will be prepared in English and Arabic. The validated Arabic-version DASS-21 and GSE were obtained from earlier studies [68, 70] and will be re-validated by an expert panel with the content validity index (CVI) [73, 74]. Two independent qualified linguistic translators who are native Arabic speakers and experts in English will evaluate and approve any parallels in the Arabic and English versions. After translation, the questionnaires will be pre‐tested by 30 Arab students from the Arabic schools excluded from the main study, to check the clarity and understandability of the items. Similarly, the validity and reliability of the Arabic Brief COPE Inventory will be checked as it has not been assessed by Arab adolescents previously.

The questionnaires will be distributed via Google Forms and will be completed in two stages. In stage one, the sociodemographic and DASS-21 questionnaires will be distributed to all students for screening. In stage two, the GSE and Brief COPE Inventory will be completed only by participants with mild to extra severe anxiety, depression, or stress scores.

#### Data analysis

The data will be analysed using SPSS 28 (IBM Corp.). A P-value of < 0.05 will indicate statistical significance. The data analysis will consider the student clustering within the schools. The baseline data will be described using descriptive analyses using the count and percentages (categorical or dichotomous variables) and the mean and standard deviation (continuous variables). To compare the sociodemographic variables and primary and secondary outcome measures between the two groups, categorical variables will be compared using the chi-square test. The independent t-test will be used to compare continuous, normally distributed variables, and the Mann-Whitney-U test will be used to compare non-normally distributed variables. The intention-to-treat (ITT) approach will be used, and all students randomised will be taken into account, regardless of their compliance [75]. Multiple imputation techniques will be applied to replace the missing data under the assumption of missing completely at random (MCAR) [76].

The intervention effectiveness will be evaluated with the Generalised Estimation Equation (GEE). Precisely, differences in the research hypothesis in terms of depression, anxiety, and stress levels will be compared at time 1 (pre-intervention), time 2 (immediately after the intervention), and time 3 (three-month follow-up) between and within the two groups adjusted for covariate variables.

The GEE method will be used due to its efficiency in suitably modelling the correlation structure of the pre-post repeated measures and its low reliance on normality assumption in the data distributions for the variables. Furthermore, multilevel models, such as the GEE, are the most appropriate for cluster RCTs as they enable the estimation of treatment effects (group differences) spanning multiple time points in a single statistical model [77, 78]. The GEE method focuses on the mean response changes over time of the entire sample and the effect of covariates on these changes [78, 79].

## Discussion

This likely is one of the first clinical trials of an online life skills intervention among Arab adolescents in Malaysia, which could be highlighted as a strategy for enhancing migrant adolescents’ life skills and improving their mental health. Due to the increasing complexity of life and especially after the Arab Spring protests, there is a significantly greater necessity for life skills education among Arab adolescent migrants than among their local counterparts [30, 80–82].

This study was designed to ameliorate and strengthen the life skills of Arab adolescents, enabling their development of protective skills against mental health issues. The empirical evidence from the study will communicate the effectiveness of life skill education as a promotion and prevention measure in mental health. Furthermore, incorporating prevention rather than cure strategies in mental health would be timely. The findings will be useful for policymakers seeking to improve the life quality of migrant adolescents with disadvantaged mental health status.

It is expected that this trial will contribute valuable insight into the application and efficacy of online life skills educational programmes for reducing anxiety, depression, and stress among Arab adolescents and improving their behaviour. This initial cluster RCT is also aimed at obtaining information on the viability of potential future full-scale trials to enhance the approach to intervention and design. The evaluation results will yield contextual data on the operational decisions of researchers, Arab student migrants, and higher education stakeholders.

Implementing such adolescent mental health programmes can enable the development of adolescent mental health services provided through the prevailing health and social services. This development would encourage organised inter-sector action for positive mental health and mental disorder prevention, thereby establishing a connection between health services and education to facilitate early identification and control of mental and psychological disorders. Furthermore, it would enable the reinforcement of health systems and promote observation, assessment, and investigation.

Apart from bridging the gap of limited access to mental health information and service for Arab adolescents, this study will aid in the reduction of mental health screening barriers for Arab students. It could increase awareness and understanding of mental health issues and the potential benefits of seeking mental health services. It could provide a supportive program for Arab students, which could help to reduce stigma and increase social support. For example, peer support activities could encourage more students to seek mental health screening and services. Moreover, it could develop and implement culturally sensitive mental health screening tools that are tailored to the specific needs and preferences of Arab students. This could help address some cultural and linguistic barriers that may prevent Arab students from seeking screening. The findings may contribute to the trustworthiness of the efficacy of a life skills intervention in teaching Arab secondary school students about mental health and how to manage daily stressors.

This research will directly benefit the students, their families, and friends and provide information to health care workers and researchers who endeavour to produce beneficial interventions for adolescent migrants. The findings will also benefit many organisations and higher authorities and can assist in implementing life skills intervention within the school curriculum of other Arab populations worldwide. The interventions are also expected to promote mental health and empower Arab adolescents with the essential life skills and resources to reach their desired abilities and overcome challenges [83].

There are some challenges in this study. Firstly, obtaining permission from all Arabic schools in the selected geographic area was difficult, which may lead to small sample size and limit the generalizability of the study. Secondly, recruiting and retaining participants, especially adolescents can be challenging in this study. Therefore, several strategies will be used to support program promotion and maintenance, such as creating a supportive and welcoming environment to address any concerns or questions for the participants at any time on the WhatsApp groups for each school, which is the most popular application in Malaysia. Provide time flexibility in completing the questionnaires and rescheduling the intervention sessions for those who missed these sessions for any reason. This will assist at the participants’ convenience to accommodate their busy schedules. The researchers will follow up with participants who have not completed the questionnaires or the intervention sessions to remind them of the importance of their participation and motivate them to complete the required tasks. The participants will get feedback on their progress, attendance, and engagement and address any barriers or concerns impacting their involvement. Researchers will also provide incentives for program attendance, such as monetary rewards, gift cards, and other meaningful rewards to the participants.

## Supporting information

S1 Checklist SPIRIT checklist. (DOC)

S1 File Study protocol. (DOC)

S1 Appendix Ethical approval letter. (DOCX)

S2 Appendix Guardian’s parent’s_consent. (DOCX)

S3 Appendix Respondent’s informed consent form. (DOCX)
