# Supplementary material for: Effect of online intervention based on life skills for mental health, self-efficacy and coping skills among Arab adolescents in the Klang Valley, Malaysia: A cluster randomised controlled trial protocol
Source: PLoS One. 2024 Feb 23;19(2):e0298627. doi: 10.1371/journal.pone.0298627 (PMC10889627; doi:10.1371/journal.pone.0298627)
Supplement: S1 File — (DOC) [file pone.0298627.s002.doc]

**1. Research title:** EFFECTIVENESS OF AN ONLINE LIFE SKILLS BASED INTERVENTION ON DEPRESSION ANXIETY AND STRESS AMONG ARABIC ADOLESCENTS IN KLANG VALLEY, MALAYSIA

**2. Principal investigator:** Dr. Ahmad Zaid Fattah Azman

**3. Co-investigators:** YOSRA MOHAMED SHERIF

**4. Introduction**

**Background of the study**

Mental health is an essential element for the well-being of all individuals, families, and societies. It is fundamental to the population's health, quality of life and social and economic stability (Moksnes & Reidunsdatter, 2019). World Health Organization (WHO) has defined mental health as "a state of well-being in which the individual realizes his or her own abilities, can cope with the normal stresses of life, can work productively and fruitfully, and is able to contribute to his or her community” (Srivastava, 2011; Galderisi et al., 2017).

Children and adolescents' mental health are a global public health issue widely recognised as a critical component of research and government intervention (Barker et al., 2019). Worldwide, it is estimated that between 10% and 20% of children and adolescents suffer from mental health problems (Barker et al., 2019; Teresa Gutmann et al., 2019).

Mental health conditions account for sixteen per cent of the global burden of disease and injury in people aged between ten to nineteen years (WHO, 2018). Half of adult mental health conditions arise in the middle of Adolescence, while three-quarters of them occurring by the middle of their twenties (WHO, 2019).

Depression and anxiety are the most frequently encountered mental health problems, which significantly impact children and adolescents. These disorders have been shown to increase the risk of adverse outcomes such as impairment, loss of productivity and community involvement, decreased educational performance, increased likelihood of engaging in risky behaviours, disability, and increased self-harm and suicide rates (Dray et al., 2015). Worldwide, depression is the fourth leading cause of illness and disability among adolescents aged 15-19 years and fifteenth for those aged 10-14 years. Meanwhile, anxiety is the ninth leading cause among 15-19 years old adolescents and sixth for those aged 10-14 years old (WOH, 2018).

Migration is also stressful for children and youth and their families during immigration and even after the settlement (Triantafyllou et al., 2018). Children of immigrants face social and emotional changes during the development stage, and they face challenges as their families adopt new cultures and languages within an unfamiliar setting. The emotional well-being of children from immigrant families is a critical public health concern. The most common mental health disorders affecting these children are manifested externally as disruptive behaviours, arguing, fighting, rule-breaking, or internally with features such as pessimistic thinking, sadness, anxiety, and loss of interest in daily activities (Kim et al., 2018).

Many studies confirm migration as a high-risk factor for mental health problems. Migration-related stress, economic disadvantages, and discrimination increase the vulnerability of the persons concerned. Migrant children and adolescents are more endangered than their native peers (Teresa Gutmann et al., 2019). Therefore, it is essential to develop and design education programmes that educate and support a diverse range of teenagers who may be at risk for developing mental health difficulties, especially those susceptible to having issues with their internalising problems (Cho, 2016).

Students spend a substantial amount of their day in school. The school setting and atmosphere are ideal for targeting all individuals, particularly those with severe symptoms who may not have sought counselling or are at risk of becoming symptomatic due to external stressors or internal vulnerabilities and those who may develop symptoms in the future (Calear et al., 2018).

School plays a vital role in developing cognitive and social skills and coping mechanisms to deal with stress. Additionally, school-based mental health programmes appear to decrease the stigma surrounding mental health and increase the frequency of consumption, especially among ethnic minorities (van Loon et al., 2020). School-based programmes eliminate many barriers, including cost, location, time, transportation, and stigmatisation (Calear et al., 2018).

Life Skills Program is one of the organized educational interventions that aim to improve mental health and boost positive and adaptive behaviour among the target individuals (UNICEF, 2015). Life skills intervention has effectively targeted different areas of adolescent health, including psychosocial, physical, and sexual and reproductive health outcomes (Singla et al., 2020). Life skills programs also play an essential part in enhancing students' academic success and non-academic areas, such as strengthening coping mechanisms and developing self-confidence and empathy (State, 2012).

Life Skills Education includes activities that support critical and creative thinking, coping with emotions and stress, self-awareness and empathy, decision-making and problem-solving, communication skills, and interpersonal relations (Nasheeda et al., 2019). Previously, online-based interventions have effectively addressed common mental health conditions among adolescents by reducing the current burden on traditional counselling services (El Morr et al., 2020) and significantly improving mental health (Spijkerman et al., 2016; Wong et al., 2014).

In this regard, this research aims to examine and determine the effect of online life skills-based intervention in reducing depression, anxiety, and stress among Arabic adolescents at Arabic schools in the Klang Valley, Malaysia, increasing their self-efficacy and coping skills.

**Problem statement and study rationale**

Migrant children and adolescents are at increased risk of anxiety and depression. The reasons for that due to asymmetric acculturation within the family, lack of adequate support from their parents and restrictive processes due to discrimination, poor socioeconomic status, living in more crowded houses and settlements in districts with insufficient infrastructure in cities and inadequate access to the healthcare system (Akkaya-Kalayci et al 2017; Atramentova et al 2018; Kim et al., 2018; Gatt, J. M.,2020; Lachal, J., et al 2020).

So, compared to the rest of the world, most Arabic populations are below 25 years (Abou Abbas & AlBuhairan, 2017). Arab adolescents bear a higher mental and behavioural disorders burden. They face additional critical challenges that can negatively affect their mental health. These include high illiteracy rates, sharp disparities in education and deterioration of education quality, inadequate health services, and poor access to health facilities, especially mental health facilities. These risk factors are further exacerbated by the dramatic changes that many Arab countries are experiencing due to severe armed conflicts, violence, and suppression (Latefa A. Dardas et al., 2016).

Among Arabic children and adolescents in The Eastern Mediterranean Region (EMR), mental disorders are estimated between10% to 36%. These figures are similar or slightly higher than the global estimate of 20% (Desouky et al., 2015). Between 1990 and 2015, there is a clear pattern toward an increasing burden of mental disorders in Arabic countries when compared to the international estimate. Conditions such as depression and anxiety account for most of the burden (Rahman, 2018), and represent a more significant percentage of disability-adjusted life years (DALYs) lost among Arab teenagers, with a high rate in armed conflict and displacement areas like Iraq, Lebanon, Algeria, and Palestine (Obermeyer, Bott, and Sassine 2015). Figure 2 illustrate that mental and behavioural disorders represented the most common cause for disability-adjusted life years (DALYs) among adolescents aged (10-19).

Palestine, for instance, had the most considerable disadvantage of mental disorders; this burden is likely to increase due to the unrest and conflict; 54.4% of Palestinian boys and 46.5% of Palestinian girls (age 6–12 years) were estimated to have mental disorders. Moreover, 37.4% of Iraqi schoolchildren were estimated to suffer from mental illnesses, and 33.5% of Syrian refugees in Turkey had Post-Traumatic Stress Disorder (Charara et al., 2017).

**Significance of the Study:**

Promoting and improving mental and emotional health is critical especially when considering migrant children and adolescents as they are part of the society and they will eventually become adults, they can engage with healthy lifestyles and reduce the burden of diseases and empower the abilities to succeed in adulthood.

This study can help Arabic students to reduce screening barriers apart from bridging the gap due to limited access to information and service for Arabic adolescents. This study also may contribute to the credibility of the effectiveness of utilizing a life skills education intervention in educating Arabic secondary school students about mental health and how to cope with daily stressors.

This research will directly benefit the students, their families, and friends, as well as to provide information to the researchers and health care workers who strive to produce beneficial interventions for adolescent migrants. Our finding also will benefit many organisations and high authorities such as Arabic embassies and Arabic school administrators, and the ministry of health and education of Arabic countries to improve mental health among Arabic children and adolescents. We expect that the interventions will promote mental health and empower teenagers with the essential life skills resources to reach the desired ability and overcome challenges.

Consequently, implementation of such programs for adolescent mental health can facilitate the development of adolescent mental health services delivered through the existing health and social services. Promote coordinated inter-sectoral action for positive mental health and prevention of mental disorders, so this will establish linkages between education and health services to enable early recognition and management of any mental, neurological and substance use disorders. Facilitate the strengthening of health systems and promote monitoring, evaluation and research.

**5. Research Question(s)**

What is the effect of online Life Skills-based intervention on depression, anxiety and stress levels, self-efficacy and coping skills between and within the intervention and control groups immediately post-intervention, and 3 months after the intervention and even after adjusted for covariate variables?

**6. Research objectives**

To determine the effect of online Life Skills based intervention on (depression, anxiety and stress), self-efficacy and coping skills among Arabic adolescents (14–18 years of age) in Klang Valley, Malaysia.

**7. Research methodology**

- **Research design**

This study is quantitative in nature and experimental in design. Specifically, this is a parallel cluster‐randomized controlled trial (cRCT). The schools will be the unit of randomization (clusters). The trial will be used to assess the effectiveness of online life skills program in reducing mental health problems (depression, anxiety and stress) among Arabic adolescents in Klang Valley, Malaysia. There are around 18 well‐known Arabic schools in Klang Valley area, with students from different Arab countries (i.e., Yemen, Iraq, Egypt, Libya, Syrian, Saudi Arabia, and etc).

- **Study location (state and justify the exact study location)**

The study will be located in Klang Valley, in the state of Selangor, Malaysia.

**Study duration:**

It is expected to be 3-4 years to complete the study from 2020-2024.

**- Study population (target population, sampling frame, sampling unit).**

**Target population**

The target population is all Arabic adolescents aged (14 -18years) at Arabic schools, in Klang Valley, Malaysia.

**Sampling Frame**

The sampling frame is a list of all eligible Arabic schools which located in Klang Valley.

**Sampling Unit**

The sampling unit of this study is the Arabic students both males and females (14-18years old) in the Arabic schools, and who will be fulfilled the inclusion and exclusion criteria during the time of the study.

- **Subject criteria** **(inclusion and exclusion criteria)**

**The inclusion criteria for schools are:**

(1) Arabic schools that agree to participate.

(2) The Arabic schools that located in Klang Valley.

**The inclusion criteria for students:**

(1) Arabic Students aged from 14 to 18 years old.

(2) students who give the assent from them and consent from their parents.

(3) student who have scored mild to extremely severe score in depression, anxiety and stress based on baseline assessment.

**The exclusion criteria for schools are:**

(1) non-Arabic schools that have Arabic students.

(2) Schools who refuse to participate in the study.

**The exclusion criteria for students:**

(1) students who refuse to participate.

(2) Students who have hearing limitations, because they cannot interact during the online session.

(3) Students who have diagnosed or treated of mental health problems before based on their school file, because it may interfere with the effect of the program.

***-* Sample size estimation (to consider non-response or attrition rate)**

**Sampling size**:

The total sample size needed will be calculated by applying the formula for two population means

Means and standard deviation used for the intervention group and control group are based on the estimation offered in a previous RCT (Osborn et al., 2020). Calculation of the required sample size in this study was performed based on the guidelines in the CONSORT for cluster RCTs. By considering the account intra-class correlation coefficient (ICC), it is necessary to multiply the sample size in RCTs by design effect=1+(m–1)*ICC, where m is the average cluster size. The formula for fixed number of clusters with unequal cluster sizes will be used. Adequate sample size after Adjust for attrition rate and expected proportion eligible and multiply with design effect= 165. Therefore, the total sample size required was 165 students. Number of clusters (schools) = estimated sample size/ cluster size = 165/54=3. Following the sample size calculation, the number of schools needed will be 3 schools. However, in order to get the required sample size, a total number of 12 schools that met the inclusion and exclusion criteria during the study time will be included in the study.

***-* Sampling method and subject recruitment**

**Sampling Method**

A cluster sampling will be used for selection of the schools. There are 12 Arabic schools in Klang Valley that meet the inclusion and exclusion criteria for this study and they will be selected to be included in the study. In order to get the required sample size, the total number of 12 schools that met the inclusion and exclusion criteria during the study time will be included in the study. Each of the schools (clusters) will then receive a serially numbered unique code. The clusters will be assigned to the intervention or control group by the ratio 1 to 1 and will be performed using block randomization using random block sizes of 2 and 4 by an independent statistician from Universiti Putra Malaysia. Therefore, 6 clusters will be in the intervention group and 6 clusters will be in the control group. Then all the students (aged 14-18 years) in the selected school, and who give assent and consent from their parents to participate will be included in the study.

**Recruitment**

For the purpose of eligibility assessment, the researcher will approach all Arabic schools in Klang Valley in Malaysia and invite these schools through formal invitation letters issued by the researcher’s university.

After getting the permission from responsible authorities of all Arabic schools (Appendix C), short online meeting will be arranged for each school. During the meeting, the researcher will explain the objectives and benefits of the study as well as the inclusion and exclusion criteria to the participants. Then a written consent will be sent to students and their parents. The students who agree to participate will be asked to answer the baseline data questionnaires (screening for eligibility). After the life skill educational intervention will be delivered to the intervention group, post-intervention assessment questionnaire will be distributed two times, immediately after the intervention completed and 3-month post-intervention for those who are eligible in screening stage.

**Randomization and Concealment Allocation**

The schools will be allocated randomly to intervention and control groups by using block randomisation after cluster size will be identified. To ensure allocation concealment, the allocation sequence of the selected schools will be assigned to a control group and an intervention group by the ratio 1 to 1 using random block sizes of 2 and 4 by an independent statistician from Universiti Putra Malaysia. To ensure allocation concealment, each cluster will receive a randomly generated treatment allocation unique code within serially numbered sealed opaque envelope. Then the research assistant will distribute the sealed opaque envelopes to the schools and the envelopes will be opened later and assigned the clusters according to their unique codes to the intervention or the control group based on the list codes generated by the software. Then the students will be assigned to the intervention group and to the control group.

***-* Research tools/instruments (state the validity/reliability/ scoring method)**

The questionnaires consist of four parts which are socio-demographic questionnaires, Depression, Anxiety, Stress Scale (DASS21), General Self-Efficacy Scale (GES), and Brief COPE scale (Appendix G). The first two parts of the questionnaires (socio-demographic and DASS21) will be sent to all students for screening purposes. The next 2 parts (GES and Brief Cope Scale) will be only filled by participants that reported mild to extra severe score of depression, anxiety or stress based on part 1.

**8- Intervention**

The interventional program's objectives will be to enhance the mental health, self-efficacy and coping mechanisms of Arabic adolescents at Arabic schools in Klang Valley in Malaysia.

**Development of the Intervention**

In this study, the educational intervention will be guided theoretically by the socio-cognitive theory. By consulting experts in this field, it will be developed based on the WHO Life Skills Education Program and UNICEF guidelines for implementing life skills.

The modules will be based on the research objectives, the target population, Arabic culture and the duration of the study, and the results of the pilot study and under the supervision of supervisory committee. The intervention will be intended to enhance the mental health and try to help participants to decrease depression, anxiety and stress, increase self-efficacy and the coping skills. This program will be also designed to improve participants’ ability to cope with the life’s challenges during migration and after settlement in the new country. some of the important considerations will be taken during developing and designing the preliminary activities, as they will be directly designed to focus on the coping with external stressors and emotions.

***-* Data collection (state the method, ensure privacy and confidentiality, quality assurance)**

At baseline before conducting the Life skill education program, questionnaires include (socio-demographic, Depression, anxiety and stress scale DASS-21, self-efficacy, and Brief Cope Scale) will be distributed to both intervention and control groups. The follow-up evaluation will be carried out immediately, and three months after the educational intervention for all students in the intervention group and the control group. The only exception being the questions on socio-demographics will be excluded for post-tests evaluation.

Data will be held in a confidential and anonymous manner ***and no third party “will have access or visibility to your data. This will be used for research purposes only***

9- **Study flowchart (diagrammatic illustration)**

Diagrammatic illustration of the study framework which provides a context to explain the study finding


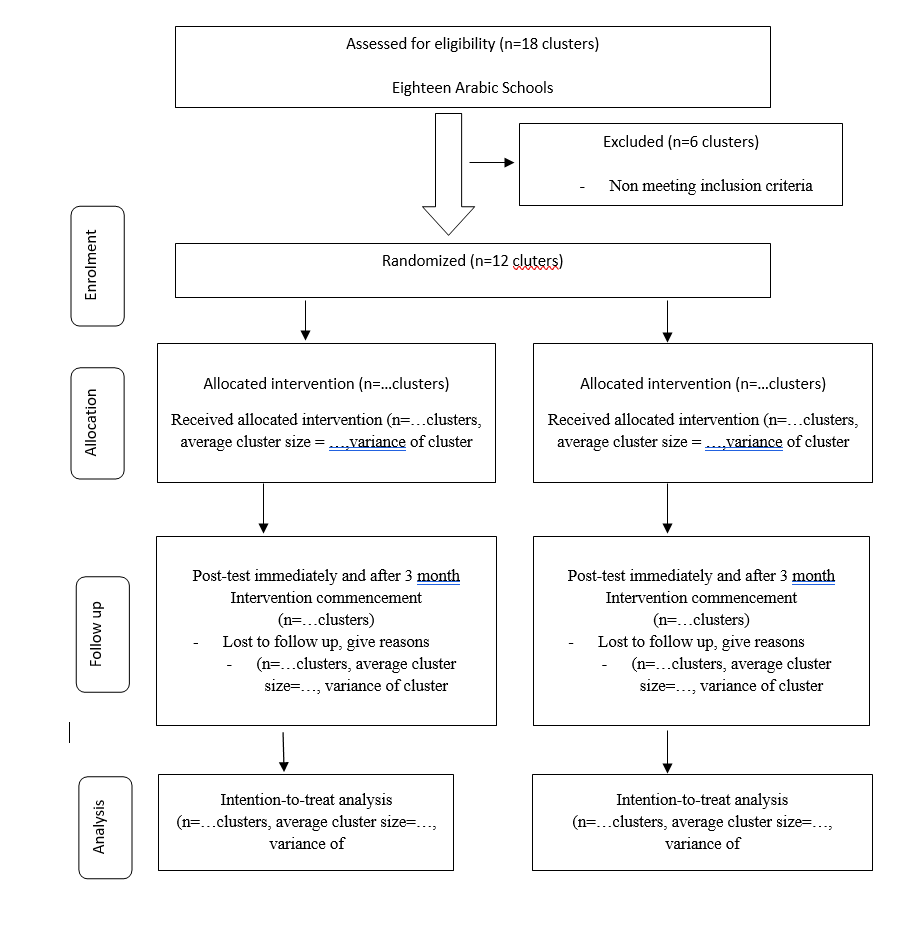


**10.** **Data analysis**

We will use SPSS Statistics version 25.0 (IBM Corp) to analyse the data. Statistical significance will be considered as a P value of less than .05. Analysis of the data will take into account clustering of students within schools. The study will use statistical analyses, including:

Descriptive analyses using the mean and standard deviation for continuous variables and the count and percentages for the dichotomous or categorical variables will be used to describe the data at baseline. Descriptive statistics of the respondents’ socio-demographic characteristics, chi-square test for nonparametric categorical variables, and the Mann-Whitney-U test was used for comparison of continuous and not normally distributed data, and 2-tailed t test for continuous variables, to compare between the control and intervention groups on socio-demographic variables and primary and secondary outcome measures. In relation to the missing data, the intention-to-treat (ITT) analysis will be used.

The effectiveness of the intervention will be assessed using the generalized estimation equation (GEE). Specifically, the differences in the research hypothesis in terms of student’s depression, anxiety and stress levels will be compared at 3 points—time 1 (pre-intervention), time 2 (immediately post-intervention), and time 3 (3-month follow-up) between and within the intervention and the control groups adjusted for covariate variables.

The GEE method will be chosen because of its efficiency in appropriately modelling the structure of the correlations of the pre-post repeated measures and its low degree of reliance on the assumption of normality in the distributions of data for the variables in the analysis. Furthermore, in the context of RCT research, multilevel models such as the GEE are the most appropriate model because they permit the estimation of treatment effects (i.e., group differences) across multiple time points within a single statistical model (Wears, 2002; Huh et al., 2012).

**11. Expected outcome**

**Primary outcome measure**

A change in the mean score of the Mental Health problems (Depression, anxiety and stress). It is expected that the education program will be effective in reducing the level of depression, anxiety and stress.

**Secondary outcome measure:**

• Increase the self-efficacy mean score and the coping skills mean score among Arabic secondary school students.

This trial is expected to provide valuable insight into the implementation and effectiveness of online Life Skills educational programs in reducing depression, anxiety and stress levels among Arabic adolescents and improving their behaviours. The aim of designing the initial cluster RCT is also to provide information regarding the feasibility of potential future full-scale trials in order to optimize the intervention and design approach. Process evaluation outcomes will provide contextual information about implementation decisions of Arabic student immigrants, researchers, and higher education stakeholders.

**12. Budget proposal [If applicable]: None**

**13.Declaration of conflict of interest: No**

**14. References**

**List all references for this study proposal**

Abd Razak, M. A., Ahmad, N. A., Abd Aziz, F. A., Jamaluddin, R., Sidik, S. M., Awaluddin, S. M., Ibrahim, N., Tan, L. A., & Sooryanarayana, R. (2019). Being Bullied Is Associated With Depression Among Malaysian Adolescents: Findings From a Cross-Sectional Study in Malaysia. Asia-Pacific Journal of Public Health, 31(8_suppl), 30S-37S. https://doi.org/10.1177/1010539519867796

Abou Abbas, O., & AlBuhairan, F. (2017). Predictors of adolescents’ mental health problems in Saudi Arabia: Findings from the Jeeluna® national study. Child and Adolescent Psychiatry and Mental Health, 11(1), 1–7. https://doi.org/10.1186/s13034-017-0188-x

Adolescent mental health. (n.d.). Retrieved August 27, 2021, from https://www.who.int/news-room/fact-sheets/detail/adolescent-mental-health

Agha, S. (2021). Mental well-being and association of the four factors coping structure model: A perspective of people living in lockdown during COVID-19. Ethics, Medicine and Public Health, 16. <https://doi.org/10.1016/j.jemep.2020.100605>

Al Omari, O., Al Sabei, S., Al Rawajfah, O., Abu Sharour, L., Aljohani, K., Alomari, K., ... & Alhalaiqa, F. (2020). Prevalence and predictors of depression, anxiety, and stress among youth at the time of COVID-19: an online cross-sectional multicountry study. Depression research and treatment, 2020.‏

Al-Yateem, N., Bani Issa, W., Rossiter, R. C., Al-Shujairi, A., Radwan, H., Awad, M., Fakhry, R., & Mahmoud, I. (2020). Anxiety related disorders in adolescents in the United Arab Emirates: A population based cross-sectional study. BMC Pediatrics, 20(1), 1–8. https://doi.org/10.1186/s12887-020-02155-0

Alegría, M. (2017). Immigration and mental health. Canada"s Mental Health, 26(2), 4–6. https://doi.org/10.1007/s40471-017-0111-2

Alenazi Shaher Falah, Hammad, S. M., & Mohamed, A. E. (2019). Prevalence of depression, anxiety and stress among male secondary school students in Arar city, Saudi Arabia, during the school year 2018 Shaher. Electronic Physician, 8(10), 3057–3061.

Alharbi, R., Alsuhaibani, K., Almarshad, A., & Alyahya, A. (2019). Depression and anxiety among high school student at Qassim Region. Journal of Family Medicine and Primary Care, 8(2). https://doi.org/10.4103/jfmpc.jfmpc_383_18

Alpaslan, A. H., Kocak, U., & Avci, K. (2016). Gender-Related Factors for Depressive Symptoms in Turkish Adolescents. Journal of Child and Adolescent Psychiatric Nursing, 29(1), 23–29. https://doi.org/10.1111/jcap.12131

Ang, A. L., Wahab, S., Abd Rahman, F. N., Hazmi, H., & Md Yusoff, R. (2019). Depressive symptoms in adolescents in Kuching, Malaysia: Prevalence and associated factors. Pediatrics International, 61(4), 404–410. https://doi.org/10.1111/ped.13778

Arabia, S. (2009). Prevalence of Depression, Anxiety and Stress as Measured by the Depression, Anxiety, and Stress Scale (DASS-42) among Secondary School Girls in Abha, Saudi Arabia. 9(August), 140–147.

Arbour-Nicitopoulos, K. P., Faulkner, G. E., & Irving, H. M. (2012). Multiple health-risk behaviour and psychological distress in adolescence. Journal of the Canadian Academy of Child and Adolescent Psychiatry, 21(3), 171–178.

Atramentova, L., Luchko, E., & Filiptsova, O. (2018). Impact of migration on the expression of aggression and empathy in urban populations. Egyptian Journal of Medical Human Genetics, 19(2), 83–86. https://doi.org/10.1016/j.ejmhg.2017.06.004

Bandura, A. (1977). Self-efficacy: Toward a Unifying Theory of Behavioral Change. Minerva Medica, 5784(2), 191–215.

Bandura, A. (1978). Social learning theory of aggression. Journal of communication, 28(3), 12-29.‏

Bandura, A. (2011). A social cognitive perspective on positive psychology. Revista de Psicología Social, 26(1), 7-20.‏

Barker, M. M., Beresford, B., Bland, M., & Fraser, L. K. (2019). Prevalence and Incidence of Anxiety and Depression among Children, Adolescents, and Young Adults with Life-Limiting Conditions: A Systematic Review and Meta-analysis. JAMA Pediatrics, 173(9), 835–844. https://doi.org/10.1001/jamapediatrics.2019.1712

Bennouna, C., Khauli, N., Basir, M., Allaf, C., Wessells, M., & Stark, L. (2019). School-based programs for Supporting the mental health and psychosocial wellbeing of adolescent forced migrants in high-income countries: A scoping review. Social Science and Medicine, 239(March), 112558. https://doi.org/10.1016/j.socscimed.2019.112558

Bettis, A. H., Coiro, M. J., England, J., Murphy, L. K., Zelkowitz, R. L., Dejardins, L., Eskridge, R., Adery, L. H., Yarboi, J., Pardo, D., & Compas, B. E. (2017). Comparison of two approaches to prevention of mental health problems in college students: Enhancing coping and executive function skills. Journal of American College Health, 65(5), 313–322. https://doi.org/10.1080/07448481.2017.1312411

Bn, A., & Ag, B. (2006). Depressive symptoms among high school adolescents in Oman. 12, 126–137.

Bryant, R. A., Edwards, B., Creamer, M., O’Donnell, M., Forbes, D., Felmingham, K. L., Silove, D., Steel, Z., Nickerson, A., McFarlane, A. C., Van Hooff, M., & Hadzi-Pavlovic, D. (2018). The effect of post-traumatic stress disorder on refugees’ parenting and their children’s mental health: a cohort study. The Lancet Public Health, 3(5), e249–e258. https://doi.org/10.1016/S2468-2667(18)30051-3

C., B., N., G., M., S., & L., W. (2016). Adverse Childhood Experiences, Resilience and Mindfulness-Based Approaches: Common Denominator Issues for Children with Emotional, Mental, or Behavioral Problems. Child and Adolescent Psychiatric Clinics of North America, 25(2), 139–156. https://doi.org/10.1016/j.chc.2015.12.001.Adverse

Calear, A. L., Christensen, H., Mackinnon, A., Griffiths, K. M., & O’Kearney, R. (2009). The YouthMood Project: A Cluster Randomized Controlled Trial of an Online Cognitive Behavioral Program With Adolescents. Journal of Consulting and Clinical Psychology, 77(6), 1021–1032. https://doi.org/10.1037/a0017391

Calear, A. L., Werner-Seidler, A., Torok, M., & Christensen, H. (2018). School-Based Prevention and Early Intervention Programs for Depression. 279–297. https://doi.org/10.1007/978-3-319-89842-1_16

Campbell, M. K., Elbourne, D. R., & Altman, D. G. (2004). CONSORT statement: Extension to cluster randomised trials. British Medical Journal, 328(7441), 702–708. https://doi.org/10.1136/bmj.328.7441.702

Ceri, V., Özlü-Erkilic, Z., Özer, Ü., Kadak, T., Winkler, D., Dogangün, B., & Akkaya-Kalayci, T. (2017). Mental health problems of second generation children and adolescents with migration background. International Journal of Psychiatry in Clinical Practice, 21(2), 142–147. https://doi.org/10.1080/13651501.2017.1291820

Charara, R., Forouzanfar, M., Naghavi, M., Moradi-Lakeh, M., Afshin, A., Vos, T., Daoud, F., Wang, H., Bcheraoui, C. El, Khalil, I., Hamadeh, R. R., Khosravi, A., Rahimi-Movaghar, V., Khader, Y., Al-Hamad, N., Obermeyer, C. M., Rafay, A., Asghar, R., Rana, S. M., … Mokdad, A. H. (2017). The burden of mental disorders in the eastern mediterranean region, 1990-2013. PLoS ONE, 12(1), 1–17. https://doi.org/10.1371/journal.pone.0169575

Cho. (2016). A Systematic Review and Meta-Analysis of School-Based Stress, Anxiety, and Depression Prevention Programs for Adolescents. Physiology & Behavior, 176(1), 100–106. https://doi.org/10.1007/s10964-019-01085-0.A

Creswell, C., Waite, P., & Hudson, J. (2020). Practitioner Review: Anxiety disorders in children and young people – assessment and treatment. Journal of Child Psychology and Psychiatry and Allied Disciplines, 61(6), 628–643. https://doi.org/10.1111/jcpp.13186

Dardas, Latefa A., Bailey, D. E., & Simmons, L. A. (2016). Adolescent Depression in the Arab Region: A Systematic Literature Review. Issues in Mental Health Nursing, 37(8), 569–585. https://doi.org/10.1080/01612840.2016.1177760

Dardas, Latefa Ali, Silva, S. G., Smoski, M. J., Noonan, D., & Simmons, L. A. (2018). The prevalence of depressive symptoms among Arab adolescents: Findings from Jordan. Public Health Nursing, 35(2), 100–108. https://doi.org/10.1111/phn.12363

Desouky, D. E. S., Ibrahem, R. A., & Omar, M. S. (2015). Prevalence and comorbidity of depression, anxiety and obsessive compulsive disorders among saudi secondary school girls, Taif area, KSA. Archives of Iranian Medicine, 18(4), 234–238. https://doi.org/015184/AIM.008

Dionigi, A., Casu, G., & Gremigni, P. (2020). Associations of self-efficacy, optimism, and empathy with psychological health in healthcare volunteers. International Journal of Environmental Research and Public Health, 17(16), 1–13. https://doi.org/10.3390/ijerph17166001

Dray, J., Bowman, J., Freund, M., Campbell, E., Hodder, R. K., Lecathelinais, C., & Wiggers, J. (2016). Mental health problems in a regional population of Australian adolescents: Association with socio-demographic characteristics. Child and Adolescent Psychiatry and Mental Health, 10(1), 1–11. https://doi.org/10.1186/s13034-016-0120-9

Dray, J., Bowman, J., Wolfenden, L., Campbell, E., Freund, M., Hodder, R., & Wiggers, J. (2015). Systematic review of universal resilience interventions targeting child and adolescent mental health in the school setting : review protocol. Systematic Reviews, 1–8. https://doi.org/10.1186/s13643-015-0172-6

Eslami, A. A., Rabiei, L., Afzali, S. M., Hamidizadeh, S., & Masoudi, R. (2016). The effectiveness of assertiveness training on the levels of stress, anxiety, and depression of high school students. Iranian Red Crescent Medical Journal, 18(1), 1–10. https://doi.org/10.5812/ircmj.21096

Felitti, V. J., Anda, R. F., Nordenberg, D., Williamson, D. F., Spitz, A. M., Edwards, V., Koss, M. P., & Marks, J. S. (2019). Relationship of Childhood Abuse and Household Dysfunction to Many of the Leading Causes of Death in Adults: The Adverse Childhood Experiences (ACE)Study. American Journal of Preventive Medicine, 56(6), 774–786. https://doi.org/10.1016/j.amepre.2019.04.001

Foo, S. Q., Tam, W. W., Ho, C. S., Tran, B. X., Nguyen, L. H., McIntyre, R. S., & Ho, R. C. (2018). Prevalence of depression among migrants: A systematic review and meta-analysis. International Journal of Environmental Research and Public Health, 15(9). <https://doi.org/10.3390/ijerph15091986>

Fusar-Poli, P. (2019). Integrated mental health services for the developmental period (0 to 25 years): A critical review of the evidence. Frontiers in Psychiatry, 10(JUN), 1–17. https://doi.org/10.3389/fpsyt.2019.00355

Galderisi, S., Andreas, H., Marianne, K., Julian, B., & Norman, S. (2017). A proposed new definition of mental health. 51(3), 407–411.

Galderisi, S., Rucci, P., Kirkpatrick, B., Mucci, A., Gibertoni, D., Rocca, P., Rossi, A., Bertolino, A., Strauss, G. P., Aguglia, E., Bellomo, A., Murri, M. B., Bucci, P., Carpiniello, B., Comparelli, A., Cuomo, A., De Berardis, D., Dell’Osso, L., Di Fabio, F., & Gelao, B. (2018). Interplay Among Psychopathologic Variables, Personal Resources, Context-Related Factors, and Real-life Functioning in Individuals With Schizophrenia: A Network Analysis. JAMA Psychiatry, 75(4), 396–404. https://doi.org/10.1001/jamapsychiatry.2017.4607

Gatt, J. M., Alexander, R., Emond, A., Foster, K., Hadfield, K., Mason-Jones, A., Reid, S., Theron, L., Ungar, M., Wouldes, T. A., & Wu, Q. (2020). Trauma, Resilience, and Mental Health in Migrant and Non-Migrant Youth: An International Cross-Sectional Study Across Six Countries. Frontiers in Psychiatry, 10(March), 1–15. https://doi.org/10.3389/fpsyt.2019.00997

González-Echevarría, A. M., Rosario, E., Acevedo, S., & Flores, I. (2019). Impact of coping strategies on quality of life of adolescents and young women with endometriosis. Journal of Psychosomatic Obstetrics and Gynecology, 40(2), 138–145. https://doi.org/10.1080/0167482X.2018.1450384

Gutmann, M. T., Aysel, M., Özlü-Erkilic, Z., Popow, C., & Akkaya-Kalayci, T. (2019). Mental health problems of children and adolescents, with and without migration background, living in Vienna, Austria. Child and Adolescent Psychiatry and Mental Health, 13(1), 1–9. https://doi.org/10.1186/s13034-019-0295-y

Hadidi, M. S., & Al Khateeb, J. M. (2015). Special Education in Arab Countries: Current challenges. International Journal of Disability, Development and Education, 62(5), 518–530. https://doi.org/10.1080/1034912X.2015.1049127

Hazell, P. (2015). Depression in children and adolescents: complementary therapies. BMJ Clinical Evidence, 2015(August 2014), 1–10. http://www.ncbi.nlm.nih.gov/pubmed/26649557%0Ahttp://www.pubmedcentral.nih.gov/articlerender.fcgi?artid=PMC4673912

Ismail, A. (2017). The prevalence and risk factors of anxiety disorders in an Egyptian sample of school and students at the age of 12–18 years. European Psychiatry, 41(S1), S410–S411. https://doi.org/10.1016/j.eurpsy.2017.01.348

Jaber, R. M., Farroukh, M., Ismail, M., Najda, J., Sobh, H., Hammad, A., & Dalack, G. W. (2015). Measuring depression and stigma towards depression and mental health treatment among adolescents in an Arab-American community. International Journal of Culture and Mental Health, 8(3), 247–254. https://doi.org/10.1080/17542863.2014.953188

Jaisoorya, T. S., Geetha, D., Beena, K. V., Beena, M., Ellangovan, K., & Thennarasu, K. (2017). Prevalence and correlates of psychological distress in adolescent students from India. East Asian Archives of Psychiatry, 27(2), 56–62.

Jamali, S., Sabokdast, S., Sharif Nia, H., Goudarzian, A. H., Beik, S., & Allen, K.-A. (2016). The Effect of Life Skills Training on Mental Health of Iranian Middle School Students: A Preliminary Study. Iranian Journal of Psychiatry, 11(4), 269–272.

James, S. L., Abate, D., Abate, K. H., Abay, S. M., Abbafati, C., Abbasi, N., Abbastabar, H., Abd-Allah, F., Abdela, J., Abdelalim, A., Abdollahpour, I., Abdulkader, R. S., Abebe, Z., Abera, S. F., Abil, O. Z., Abraha, H. N., Abu-Raddad, L. J., Abu-Rmeileh, N. M. E., Accrombessi, M. M. K., … Murray, C. J. L. (2018). Global, regional, and national incidence, prevalence, and years lived with disability for 354 diseases and injuries for 195 countries and territories, 1990–2017: a systematic analysis for the Global Burden of Disease Study 2017. The Lancet, 392(10159), 1789–1858. https://doi.org/10.1016/S0140-6736(18)32279-7

Johnson, S. E., Lawrence, D., Hafekost, J., Saw, S., Buckingham, W. J., Sawyer, M., Ainley, J., & Zubrick, S. R. (2016). Service use by Australian children for emotional and behavioural problems: Findings from the second Australian Child and Adolescent Survey of Mental Health and Wellbeing. Australian and New Zealand Journal of Psychiatry, 50(9), 887–898. https://doi.org/10.1177/0004867415622562

Johnson, S. E., Lawrence, D., Sawyer, M., & Zubrick, S. R. (2018). Mental disorders in Australian 4- to 17- year olds: Parent-reported need for help. Australian & New Zealand Journal of Psychiatry, 52(2), 149–162. http://10.0.4.153/0004867417706032

Kaabi, N. Al, Aziz Selim, N. A., Singh, R., Almadahki, H., & Salem, M. (2017). Prevalence and Determinants of Depression among Qatari Adolescents in Secondary Schools. Family Medicine & Medical Science Research, 06(03). https://doi.org/10.4172/2327-4972.1000219

Kann, L. (2019). Youth Risk Behavior Surveillance — United States, 2017. MMWR. Surveillance Summaries, 67(8), 1–114. https://doi.org/10.15585/MMWR.SS6708A1

Kato, T. (2015). Frequently used coping scales: A meta-analysis. Stress and Health, 31(4), 315–323. https://doi.org/10.1002/smi.2557

Khalid, A., Qadir, F., Chan, S. W. Y., & Schwannauer, M. (2019). Adolescents’ mental health and well-being in developing countries: a cross-sectional survey from Pakistan. Journal of Mental Health, 28(4), 389–396. https://doi.org/10.1080/09638237.2018.1521919

Kien, C., Sommer, I., Faustmann, A., Gibson, L., Schneider, M., Krczal, E., Jank, R., Klerings, I., Szelag, M., Kerschner, B., Brattström, P., & Gartlehner, G. (2019). Prevalence of mental disorders in young refugees and asylum seekers in European Countries: a systematic review. European Child and Adolescent Psychiatry, 28(10), 1295–1310. https://doi.org/10.1007/s00787-018-1215-z

Kim, H.-J., Lee, E.-H., Hwang, S.-T., Hong, S.-H., & Kim, J.-H. (2018). Psychometric Properties of the Children’s Depression Inventory-2 among a Community-Based Sample of Korean Children and Adolescents. In Korean Journal of Clinical Psychology (Vol. 37, Issue 2, pp. 178–187). https://doi.org/10.15842/kjcp.2018.37.2.005

Kim, J. H., Nicodimos, S., Kushner, S. E., Rhew, I. C., McCauley, E., & Vander Stoep, A. (2018). Comparing Mental Health of US Children of Immigrants and Non-Immigrants in 4 Racial/Ethnic Groups. Journal of School Health, 88(2), 167–175. https://doi.org/10.1111/josh.12586

Klassen, R. M., & Klassen, J. R. L. (2018). Self-efficacy beliefs of medical students: a critical review. Perspectives on Medical Education, 7(2), 76–82. https://doi.org/10.1007/s40037-018-0411-3

Lachal, J., Moro, M. R., Carretier, E., Simon, A., Barry, C., Falissard, B., & Rouquette, A. (2020). Assessment of transcultural psychotherapy to treat resistant major depressive disorder in children and adolescents from migrant families: Protocol for a randomized controlled trial using mixed method and Bayesian approaches. International Journal of Methods in Psychiatric Research, 29(4), 1–10. https://doi.org/10.1002/mpr.1847

Lee, M.-J., Wu, W.-C., Chang, H.-C., Chen, H.-J., Lin, W.-S., Feng, J. Y., & Lee, T. S.-H. (2020). Effectiveness of a school-based life skills program on emotional regulation and depression among elementary school students: A randomized study. Children & Youth Services Review, 118, N.PAG-N.PAG. https://doi.org/10.1016/j.childyouth.2020.105464

Maalouf, F. T., Ghandour, L. A., Halabi, F., Zeinoun, P., Shehab, A. A. S., & Tavitian, L. (2016). Psychiatric disorders among adolescents from Lebanon: prevalence, correlates, and treatment gap. Social Psychiatry and Psychiatric Epidemiology, 51(8), 1105–1116. https://doi.org/10.1007/s00127-016-1241-4

Malak, M. Z., & Khalifeh, A. H. (2018). Anxiety and depression among school students in Jordan: Prevalence, risk factors, and predictors. Perspectives in Psychiatric Care, 54(2), 242–250. https://doi.org/10.1111/ppc.12229

MARJAN MOHAMMADZADEH, and Suriani Binti Ismail Hamidin Bin Awang, Hayati Binti Kadir. 2017. “EFFECTS OF LIFE SKILLS EDUCATION ON EMOTIONAL PROBLEMS, SELF-ESTEEM AND COPING MECHANISMS AMONG INSTITUTIONAL ADOLESCENTS IN KLANG VALLEY, MALAYSIA.” UPM THESIS 10(January):1–27.

McLeroy, L. L. & K. R. (2021). Self_efficacy_and_Health_Education.pdf. The Journal of School Health, 56(8), 317–321.

McMullen, J. D., & McMullen, N. (2018). Evaluation of a teacher-led, life-skills intervention for secondary school students in Uganda. Social Science & Medicine, 217, 10–17. http://10.0.3.248/j.socscimed.2018.09.041

Miller, K., Koppenol-Gonzalez, G., Jawad, A., Steen, F., Sassine, M., & Jordans, M. (2020). A Randomised Controlled Trial of the I-Deal Life Skills Intervention with Syrian Refugee Adolescents in Northern Lebanon. Intervention, 18(2), 119–128. https://doi.org/10.4103/INTV.INTV_4_20

Mirkin, B. (2010). Population levels, trends and policies in the Arab Region: Challenges and opportunities, Arab Human Development Report. United Nations Development Programme, 1–41.

Moeini, B., Bashirian, S., Soltanian, A. R., Ghaleiha, A., & Taheri, M. (2019). Prevalence of depression and its associated sociodemographic factors among Iranian female adolescents in secondary schools. BMC Psychology, 7(1), 1–11. https://doi.org/10.1186/s40359-019-0298-8

Mohammadi, M. R., Alavi, S. S., Ahmadi, N., Khaleghi, A., Kamali, K., Ahmadi, A., Hooshyari, Z., mohamadian, F., Jaberghaderi, N., Nazaribadie, M., sajedi, Z., Farshidfar, Z., Kaviani, N., Davasazirani, R., Shahbakhsh, A. J., Rad, M. R., shahbazi, K., Khodaverdloo, R. R., Tehrani, L. N., … Ashoori, S. (2019). The prevalence, comorbidity and socio-demographic factors of depressive disorder among Iranian children and adolescents: To identify the main predictors of depression. Journal of Affective Disorders, 247(January), 1–10. https://doi.org/10.1016/j.jad.2019.01.005

Mohammadzadeh, M, Awang, H., Ismail, S., & HK, S. (2020). Improving coping mechanisms of Malaysian adolescents living in orphanages through a life skills education program: a multicentre randomized controlled trial. Asian Journal of Psychiatry, 48. https://doi.org/10.1016/j.ajp.2019.101892

Mohammadzadeh, Marjan, Awang, H., Ismail, S., & Shahar, H. K. (2019). Improving emotional health and self-esteem of Malaysian adolescents living in control trial Improving emotional health and self-esteem of Malaysian adolescents living in orphanages through Life Skills Education program : A multi-centre randomized control . December. https://doi.org/10.1371/journal.pone.0226333

Mojtabai, R., Olfson, M., & Han, B. (2016). National trends in the prevalence and treatment of depression in adolescents and young adults. Pediatrics, 138(6). https://doi.org/10.1542/peds.2016-1878

Moksnes, U. K., & Reidunsdatter, R. J. (2019). Self-esteem and mental health in adolescents - level and stability during a school year. Norsk Epidemiologi, 28(1–2), 59–67. <https://doi.org/10.5324/nje.v28i1-2.3052>

Mpaata, K. A. (2008). Aggressive behaviour in education institutions: theoretical perspectives and implications for school and university managers in Uganda. Retrieved on April, 3, 2015.‏

Muris, P., Simon, E., Lijphart, H., Bos, A., Hale, W., Schmeitz, K., Albano, A. M., Bar-Haim, Y., Beesdo-Baum, K., Beidel, D., Bender, P., Borelli, J., Broeren, S., Cartwright-Hatton, S., Craske, M., Crawford, E., Creswell, C., DeSousa, D., Dodd, H., … Wolters, L. (2017). The Youth Anxiety Measure for DSM-5 (YAM-5): Development and First Psychometric Evidence of a New Scale for Assessing Anxiety Disorders Symptoms of Children and Adolescents. Child Psychiatry and Human Development, 48(1), 1–17. https://doi.org/10.1007/s10578-016-0648-1

Nabavi, R. T. (2012). Theories of Developmental Psychology: Bandura ’ s Social Learning Theory & Social Cognitive Learning Theory. Research Gate, January 2012, 1–24.

Ndetei, D. M., Mutiso, V., Gitonga, I., Agudile, E., Tele, A., Birech, L., Musyimi, C., & McKenzie, K. (2019). World Health Organization life-skills training is efficacious in reducing youth self-report scores in primary school going children in Kenya. Early Intervention in Psychiatry, 13(5), 1146–1154. https://doi.org/10.1111/eip.12745

Obermeyer, C. M., Bott, S., & Sassine, A. J. (2015). Arab Adolescents: Health, Gender, and Social Context. Journal of Adolescent Health, 57(3), 252–262. https://doi.org/10.1016/j.jadohealth.2015.01.002

Oderinde, K. O., Dada, M. U., Ogun, O. C., Awunor, N. S., Awunor, N. S., Ahmed, H. K., Tsuung, A. B., Tanko, S. T., & Yusuff, A. A. (2018). Prevalence and Predictors of Depression among Adolescents in Ido Ekiti, South West Nigeria. International Journal of Clinical Medicine, 09(03), 187–202. https://doi.org/10.4236/ijcm.2018.93017

Osborn, T. L., Wasil, A. R., Venturo-Conerly, K. E., Schleider, J. L., & Weisz, J. R. (2020). Group Intervention for Adolescent Anxiety and Depression: Outcomes of a Randomized Trial with Adolescents in Kenya. Behavior Therapy, 51(4), 601–615. https://doi.org/10.1016/j.beth.2019.09.005

Pampati, S., Alattar, Z., Cordoba, E., Tariq, M., & Leon, C. M. De. (2018). Mental health outcomes among Arab refugees , immigrants , and U . S . born Arab Americans in Southeast Michigan : a cross- sectional study. 1–9.

Pannebakker, F. D., van Genugten, L., Diekstra, R. F. W., Gravesteijn, C., Fekkes, M., Kuiper, R., & Kocken, P. L. (2019). A Social Gradient in the Effects of the Skills for Life Program on Self-Efficacy and Mental Wellbeing of Adolescent Students. Journal of School Health, 89(7), 587–595. https://doi.org/10.1111/josh.12779

Pengpid, S., & Peltzer, K. (2020a). High psychological distress among school-going adolescents in Afghanistan: prevalence and correlates from a national survey. Vulnerable Children and Youth Studies, 15(1), 40–47. https://doi.org/10.1080/17450128.2019.1679937

Pengpid, S., & Peltzer, K. (2020b). Psychological Distress and Its Associated Factors Among School-Going Adolescents in Tanzania. Psychological Studies, 65(2), 174–181. https://doi.org/10.1007/s12646-020-00550-2

Pengpid, S., & Peltzer, K. (2020c). Prevalence and associated factors of psychological distress among a national sample of in-school adolescents in Morocco. BMC Psychiatry, 20(1), 475. https://doi.org/10.1186/s12888-020-02888-3

Perreira, K. M., & Telles, E. E. (2014). The color of health: Skin color, ethnoracial classification, and discrimination in the health of Latin Americans. Social Science and Medicine, 116, 241–250. https://doi.org/10.1016/j.socscimed.2014.05.054

Plass-Christl, A., Haller, A. C., Otto, C., Barkmann, C., Wiegand-Grefe, S., Hölling, H., Schulte-Markwort, M., Ravens-Sieberer, U., & Klasen, F. (2017). Parents with Mental health problems and their children in a German population based sample: Results of the BELLA study. PLoS ONE, 12(7), 1–14. https://doi.org/10.1371/journal.pone.0180410

Pocock, L., Abyad, A., Georgy, E. E., Carr, E. C. J., Breen, A. C., Gilany, A. El, & Gilany, A. El. (2014). Child and adolescent mental health in the Middle East : an overview Editorial A feasibility study Infant feeding in Al-Hassa , Saudi Arabia Ingrowing toe nail : conservative treatment Case Report : Stroke secondary to an unusual cause. June.

Polanczyk, G. V., Salum, G. A., Sugaya, L. S., Caye, A., & Rohde, L. A. (2015). Annual research review: A meta-analysis of the worldwide prevalence of mental disorders in children and adolescents. Journal of Child Psychology and Psychiatry and Allied Disciplines, 56(3), 345–365. https://doi.org/10.1111/jcpp.12381

Possomato-Vieira, José S. and Khalil, R. A. K. (2016). HHS Public Access. Physiology & Behavior, 176(12), 139–148. https://doi.org/10.1007/s11920-018-0969-9.Social

Quispe-Tintaya, W. (2017). Prevention of Depression in At-Risk Adolescents: Predictors and Moderators of Acute Effects. Physiology & Behavior, 176(3), 139–148. https://doi.org/10.1016/j.jaac.2015.12.015.Prevention

Rahman, A. (2018). Mental disorders in the Eastern Mediterranean Region. International Journal of Public Health, 63(s1), 9–10. https://doi.org/10.1007/s00038-017-0986-1

Raknes, S., Pallesen, S., Bjaastad, J. F., Wergeland, G. J., Hoffart, A., Dyregrov, K., Håland, Å. T., & Haugland, B. S. M. (2017). Negative Life Events, Social Support, and Self-Efficacy in Anxious Adolescents. Psychological Reports, 120(4), 609–626. https://doi.org/10.1177/0033294117699820

Reem M. Ghandour, Laura J. Sherman, Catherine J. Vladutiu, Mir M. Ali, PhD2, Sean E. Lynch, LCSW2, Rebecca H. Bitsko, S. J. B. (2017). Prevalence and Treatment of Depression, Anxiety, and Conduct Problems in US Children. Physiology & Behavior, 176(3), 139–148. https://doi.org/10.1016/j.jpeds.2018.09.021.Prevalence

Richards, D. (2011). Prevalence and clinical course of depression: A review. Clinical Psychology Review, 31(7), 1117–1125. https://doi.org/10.1016/j.cpr.2011.07.004

Roy, K., Kamath, V. G., Kamath, A., Hegde, A., Alex, J., & Ashok, L. (2016). Effectiveness of Life Skill Training Program on Stress among Adolescents at a School Setting. Journal of Indian Association for Child & Adolescent Mental Health, 12(4), 309–322. http://search.ebscohost.com/login.aspx?direct=true&db=a9h&AN=118548036&site=ehost-live

S. K., V. K., & Krishnamurthy, A. R. (2016). The impact of life skills training on psychological well-being: The role of gender differences. Indian Journal of Health & Wellbeing, 7(2), 193–197. http://search.ebscohost.com/login.aspx?direct=true&db=a9h&AN=115097095&site=ehost-live

Sahril, N., Ahmad, N. A., Idris, I. B., Sooryanarayana, R., & Abd Razak, M. A. (2021). Factors Associated with Mental Health Problems among Malaysian Children: A Large Population-Based Study. Children, 8(2), 119. https://doi.org/10.3390/children8020119

Samples, N., Diagnoses, M., Salk, R. H., Hyde, J. S., Abramson, L. Y., Hyde, J. S., & Abramson, L. Y. (2017). Psychological Bulletin Gender Differences in Depression in Representative National Samples : Meta-Analyses of Diagnoses and Symptoms. 143(8), 783–822.

Sandal, R., Goel, N., Sharma, M., Bakshi, R., Singh, N., & Kumar, D. (2017). Prevalence of depression, anxiety and stress among school going adolescent in Chandigarh. Journal of Family Medicine and Primary Care, 6(2), 405. https://doi.org/10.4103/2249-4863.219988

Scholz, U., Doña, B. G., Sud, S., & Schwarzer, R. (2002). Is general self-efficacy a universal construct? Psychometric findings from 25 countries. European Journal of Psychological Assessment, 18(3), 242–251. https://doi.org/10.1027//1015-5759.18.3.242

Schwarzer, R., & Jerusalem, M. (1995). Generalized Self-efficacy Scale [J. Weinman, S. Wright, & M. Johnston]. Measures in Health Psychology: A User’s Portfolio. Causal and Control Beliefs, 2008, 35–37.

Sendi, I., Chouikh, A., Ammar, A., & Bouafia, N. (2018). Depression in a sample of Tunisian adolescents: Prevalence, associated factors and comorbidity with anxiety disorders. International Journal of Adolescent Medicine and Health, 1–10. https://doi.org/10.1515/ijamh-2018-0068

Shah, S. M., Dhaheri, F. Al, Albanna, A., Jaberi, N. Al, Eissaee, S. Al, Alshehhi, N. A., Al Shamisi, S. A., Al Hamez, M. M., Abdelrazeq, S. Y., Grivna, M., & Betancourt, T. S. (2020). Self-esteem and other risk factors for depressive symptoms among adolescents in United Arab Emirates. PLoS ONE, 15(1), 1–17. https://doi.org/10.1371/journal.pone.0227483

Shi, L., Chen, W., Bouey, J. H., Lin, Y., & Ling, L. (2019). Impact of acculturation and psychological adjustment on mental health among migrant adolescents in Guangzhou, China: A cross-sectional questionnaire study. BMJ Open, 9(5), 1–12. https://doi.org/10.1136/bmjopen-2018-022712

Silva, S. A., Silva, S. U., Ronca, D. B., Gonçalves, V. S. S., Dutra, E. S., & Carvalho, K. M. B. (2020). Common mental disorders prevalence in adolescents: A systematic review and metaanalyses. PLoS ONE, 15(4), 1–20. https://doi.org/10.1371/journal.pone.0232007

Singla, D. R., Waqas, A., Hamdani, S. U., Suleman, N., Zafar, S. W., Zill-E-Huma, Saeed, K., Servili, C., & Rahman, A. (2020). Implementation and effectiveness of adolescent life skills programs in low- and middle-income countries: A critical review and meta-analysis. Behaviour Research and Therapy, 130, 103402. https://doi.org/10.1016/j.brat.2019.04.010

Siziya, S., & Mazaba, M. L. (2015). Prevalence and Correlates for Psychosocial Distress Among In-School Adolescents in Zambia. Frontiers in Public Health, 3(July), 1–7. https://doi.org/10.3389/fpubh.2015.00180

Srivastava, K. (2011). Positive mental health and its relationship with resilience. Industrial Psychiatry Journal, 20(2), 75. https://doi.org/10.4103/0972-6748.102469

State, G. (2012). Life Skills Training as an Effective Intervention Strategy to Reduce Stress among Tibetan Refugee Adolescents. 25(4). https://doi.org/10.1093/jrs/fer056

Steel, J. L., Dunlavy, A. C., Harding, C. E., & Theorell, T. (2017). The Psychological Consequences of Pre-Emigration Trauma and Post-Migration Stress in Refugees and Immigrants from Africa. Journal of Immigrant and Minority Health, 19(3), 523–532. https://doi.org/10.1007/s10903-016-0478-z

Tolentino, J. C., & Schmidt, S. L. (2018). DSM-5 criteria and depression severity: Implications for clinical practice. Frontiers in Psychiatry, 9(OCT), 1–9. https://doi.org/10.3389/fpsyt.2018.00450

Triantafyllou, K., Othiti, I., Xylouris, G., Moulla, V., Ntre, V., Kovani, P., Gertsou, I., & Anagnostopoulos, D. (2018). Mental health and psychosocial factors in young refugees, immigrants and Greeks: A retrospective study. Psychiatrike = Psychiatriki, 29(3), 231–239. https://doi.org/10.22365/jpsych.2018.293.231

UNICEF. (2015). https://www.unicef.org/azerbaijan.

UNICEF. (2019). Comprehensive Life Skills Framework.

(UNICEF), U. N. C. F. (2016). Review of the Education Program Life.

van Loon, A. W. G., Creemers, H. E., Beumer, W. Y., Okorn, A., Vogelaar, S., Saab, N., Miers, A. C., Westenberg, P. M., & Asscher, J. J. (2020). Can Schools Reduce Adolescent Psychological Stress? A Multilevel Meta-Analysis of the Effectiveness of School-Based Intervention Programs. Journal of Youth and Adolescence, Hofferth 2009. https://doi.org/10.1007/s10964-020-01201-5

Van Loon, A. W. G., Creemers, H. E., Vogelaar, S., Saab, N., Miers, A. C., Westenberg, P. M., & Asscher, J. J. (2019). The effectiveness of school-based skills-training programs promoting mental health in adolescents: A study protocol for a randomized controlled study. BMC Public Health, 19(1), 1–12. https://doi.org/10.1186/s12889-019-6999-3

Vibhakar, V., Allen, L. R., Gee, B., & Meiser-Stedman, R. (2019). A systematic review and meta-analysis on the prevalence of depression in children and adolescents after exposure to trauma. Journal of Affective Disorders, 255(October 2018), 77–89. https://doi.org/10.1016/j.jad.2019.05.005

Werner-Seidler, A., Perry, Y., Calear, A. L., Newby, J. M., & Christensen, H. (2017). School-based depression and anxiety prevention programs for young people: A systematic review and meta-analysis. Clinical Psychology Review, 51, 30–47. https://doi.org/10.1016/j.cpr.2016.10.005

WHO. (1994). Programme on Mental Health: Life Skills in Schools. WHO World Health Organization.

WHO. (2017). Depression and other common mental disorders: global health estimates. World Health Organization, (enero, 2017), 1–24.

WHO. (2019). Adolescent Mental Health: Time for Action. 8. https://www.who.int/pmnch/knowledge/publications/AMH.pdf?ua=1

WHO. (2021). Adolescent and young adult health. https://www.who.int/news-room/fact-sheets/detail/adolescents-health-risks-and-solutions

WOH. (2020). Adolescent mental health. 28 September 2020. https://www.who.int/news-room/fact-sheets/detail/adolescent-mental-health

Wong, W. C. W., Sun, W. H., Chia, S. M. C., Tucker, J. D., Mak, W. P. H., Song, L., Choi, K. W. Y., Lau, S. T. H., & Wan, E. Y. F. (2020). Effectiveness of a Peer-Led Web-Based Intervention to Improve General Self-Efficacy in Using Dating Apps among Young Adults: Randomized Clustered Trial. Journal of Medical Internet Research, 22(10), 1–16. https://doi.org/10.2196/16378

Wood, R., & Bandura, A. (1989). Social Cognitive Theory of Organizational Management. In Academy of Management Review (Vol. 14, Issue 3, pp. 361–384). https://doi.org/10.5465/amr.1989.4279067

Wu, C. Y., & Lee, T. S. H. (2020). Impact of parent–child relationship and sex on trajectories of children internalizing symptoms. Journal of Affective Disorders, 260(July 2019), 167–173. https://doi.org/10.1016/j.jad.2019.09.016

Yeh, K. H., Bedford, O., Wu, C. W., Wang, S. Y., & Yen, N. S. (2017). Suppression benefits boys in Taiwan: The relation between gender, emotional regulation strategy, and mental health. Frontiers in Psychology, 8(FEB). https://doi.org/10.3389/fpsyg.2017.00135

Zarafshan, H., Mohammadi, M. R., & Salmanian, M. (2015). Prevalence of anxiety disorders among children and adolescents in Iran: A systematic review. Iranian Journal of Psychiatry, 10(1), 1–7.
