# Supplementary material for: Effect of online intervention based on life skills for mental health, self-efficacy and coping skills among Arab adolescents in the Klang Valley, Malaysia: A cluster randomised controlled trial protocol
Source: PLoS One. 2024 Feb 23;19(2):e0298627. doi: 10.1371/journal.pone.0298627 (PMC10889627; doi:10.1371/journal.pone.0298627)
Supplement: S1 Appendix — (DOCX) [file pone.0298627.s003.docx]

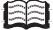

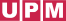


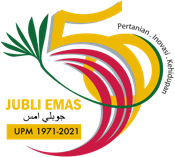

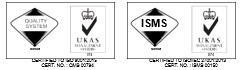


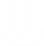

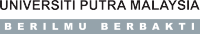

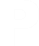

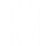


PEJABAT TIMBALAN NAIB CANSELOR (PENYELIDIKAN DAN INOVASI)

*OFFICE OF THE DEPUTY VICE CHANCELLOR (RESEARCH AND INNOVATION*

*Rujukan kami* : UPM/TNCPI/RMC/1.4.18.2 (JKEUPM)

*Tarikh* : 12 May 2022

Dr. Ahmad Zaid Fattah Bin Azman Department of Community Health Faculty of Medicine and Health Sciences Universiti Putra Malaysia

Serdang, Selangor Dear Madam/Sir,

# RESEARCH PROJECT: EFFECTIVENESS OF AN ONLINE LIFE SKILLS BASED INTERVENTION ON DEPRESSION, ANXIETY AND STRESS AMONG ARABIC ADOLESCENTS IN KLANG VALLEY, MALAYSIA.

**REFERENCE NO : JKEUPM-2021-912 RESEARCHER : YOSRA MOHAMED SHERIF**

# SUPERVISOR : DR. AHMAD ZAID FATTAH BIN AZMAN

The Ethics Committee for Research involving Human Subjects of University Putra Malaysia (JKEUPM) has studied the proposal for the above project and found that there were no objectionable ethical issues involved in the proposed study.

Please find the list of documents received and reviewed with reference to the study and committee members who reviewed the documents (as attached).

Notwithstanding above, we will not be responsible for any misconduct on the part of researcher in the course of carrying out the research.

# Ethical approval is required in the case of amendments/ changes to the study documents/ study sites/ study team.

Thank you.

# “WITH KNOWLEDGE WE SERVE”

Sincerely yours,


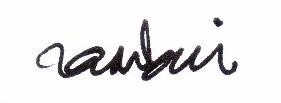


# PROF. DR. ZAMBERI SEKAWI

Chair

Ethics Committee for Research involving Human Subjects Universiti Putra Malaysia

PejabatTimbalan Naib Canselor (Penyelidikan dan Inovasi), Universiti Putra Malaysia, 43400 UPM Serdang, Selangor Darul Ehsan, Malaysia PejabatTimbalan Naib Canselor (P&I) 603-89471002 : 603-8945 1646, PejabatPentadbiran TNCPI 603-89471608 603-8945 1673, PejabatPengarah, Pusat PengurusanPenyelidikan (RMC) 603-8947 1601603-8945 1596,PejabatPengarah, Putra Science Park(PSP)

603-8947 1291 603-8946 4121 [http://www.tncpi.upm.edu.my,](http://www.tncpi.upm.edu.my/)
