# Supplementary material for: Effect of online intervention based on life skills for mental health, self-efficacy and coping skills among Arab adolescents in the Klang Valley, Malaysia: A cluster randomised controlled trial protocol
Source: PLoS One. 2024 Feb 23;19(2):e0298627. doi: 10.1371/journal.pone.0298627 (PMC10889627; doi:10.1371/journal.pone.0298627)
Supplement: S2 Appendix — (DOCX) [file pone.0298627.s004.docx]

**GUARDIAN’S/PARENT’S CONSENT**

I …………………………………… Identity Card No. …………………………… address………………………………………………………………………………………………………... ……………………………………………………..hereby voluntarily agree to allow my *son / daughter / ward....................................................................... to take part in the research stated above *(clinical/ questionnaire/drug trial/video recording/ focus group/interview).

I have been informed about the nature of the research in terms of methodology, possible adverse effects and complications (as written in the Respondent’s Information Sheet). I understand that my *son / daughter / ward has the right to withdraw from this research at any time without giving any reason whatsoever. I also understand that this study is confidential and all information provided with regard to the identity of my* son / daughter / ward will remain private and confidential.

I* wish / do not wish to know the results related to my my *son’s / daughter’s / ward’s participation in the research

I agree/do not agree that the images/photos/video recordings/voice recordings related to my son/daughter/ward be used in any form of publication or presentation. (if applicable).

* delete where necessary

Signature ……..………………………… Signature ……..………………………….....

(*Parent/Guardian*) (*Witness*)

Date :………………………………….….. Name :………………………………….…..

I/C No. :………………………………….…..

Signature ……..…………………………

(*Student*)

Date :………………………………….…..

I confirm that I have explained to the respondent’s parent/guardian the nature and purpose of the above-mentioned research.

Date ……..………………………… Signature ……..………………………….

(*Researcher*)
