# Supplementary material for: Effect of online intervention based on life skills for mental health, self-efficacy and coping skills among Arab adolescents in the Klang Valley, Malaysia: A cluster randomised controlled trial protocol
Source: PLoS One. 2024 Feb 23;19(2):e0298627. doi: 10.1371/journal.pone.0298627 (PMC10889627; doi:10.1371/journal.pone.0298627)
Supplement: S3 Appendix — (DOCX) [file pone.0298627.s005.docx]

**RESPONDENT’S INFORMED CONSENT FORM**

I …………………………………… Identity Card No. …………………………… address………………………………………………………………………………………………………... ……………………………………………………..hereby voluntarily agree to take part in the research stated above *(clinical /drug trial/video recording/ focus group/interview-based/ questionnaire-based).

I have been informed about the nature of the research in terms of methodology, possible adverse

effects and complications (as written in the Respondent’s Information Sheet). I understand that I have the right to withdraw from this research at any time without giving any reason whatsoever. I also understand that this study is confidential and all information provided with regard to my identity will remain private and confidential.

I* wish / do not wish to know the results related to my participation in the research

I agree/do not agree that the images/photos/video recordings/voice recordings related to me be used in any form of publication or presentation (if applicable)

* delete where necessary

Signature ……..………………………… Signature ……..………………………….

(Respondent) (Witness)

Date :………………………………….….. Name :………………………………….…..

I/C No. :………………………………….…..

I confirm that I have explained to the respondent the nature and purpose of the above-mentioned research.

Date ……..………………………… Signature ……..………………………….

(Researcher)
